# Anionic Bottom-Up Flux Orchestrated via Hard Carbon Surface Chemistry for Stable Sodium-Ion Batteries

**DOI:** 10.1007/s40820-026-02339-w

**Published:** 2026-09-03

**Authors:** Peiyao Wang, Shendong Xu, Siya Wang, Xiaoyu Cui, Jin Bai, Yuping Sun, Xuebin Zhu, Bangchuan Zhao, Shulei Chou, Xingqiao Wu

**Affiliations:** 1 Key Laboratory of Materials Physics, Institute of Solid State Physics, HFIPS, Chinese Academy of Sciences, Hefei, 230031 People’s Republic of China; 2 School of Carbon Neutrality Science and Engineering, Anhui University of Science and Technology, Hefei, 231131 People’s Republic of China; 3 Institute for Carbon Neutralization Technology, College of Chemistry and Materials Engineering, Wenzhou University, Wenzhou, Zhejiang 325035 People’s Republic of China

**Keywords:** Hard carbon, Sodium-ion battery, Interphase engineering, Bottom-up anionic flux, Inorganic-rich SEI

## Abstract

Hard carbon anodes for sodium-ion batteries suffer from unstable solid-electrolyte interphase formation, leading to low initial Coulombic efficiency and poor cycle stability. Herein, we demonstrate a molecular-level surface design that actively orchestrates an anionic bottom-up flux from the electrolyte bulk to the hard carbon interface. By engineering a synergistic pair of pyridinic-N and carbonyl groups on the carbon surface, we create a functional-differentiation microenvironment where pyridinic-N selectively anchors PF_6_^−^ anions, while the cooperative carbonyl repels solvent molecules. This dual functionality establishes a sustained concentration gradient that drives a continuous reverse flux of anions toward the interface, fundamentally redirecting the electrolyte decomposition pathway from solvent-dominated to anion-preferential. The tailored surface lowers the PF_6_^−^ decomposition barrier by over 70%, yielding a thin, inorganic-rich solid-electrolyte interphase dominated by NaF and Na_2_O. The optimized anode achieves 91.9% Coulombic efficiency with high reversible capacity of 368.2 mAh g^−1^, and 96.5% capacity retention after 5,000 cycles. A pouch cell assembled with NFPP cathode achieves an energy density of 239.1 Wh kg^−1^ and stable operation over 500 cycles, demonstrating strong practical potential. This work establishes active surface-guided anionic transport as a powerful strategy for interphase engineering in advanced sodium-ion batteries.

**Supplementary Information:**

The online version contains supplementary material available at https://doi.org/10.1007/s40820-026-02339-w.

## Highlights

- A cooperative pair of pyridinic-N and carbonyl groups creates a trap-and-repel microenvironment that actively programs interfacial reactions.
- The functional differentiation drives a sustained bottom-up anionic flux, steering decomposition toward an anion-derived pathway.
- The resulting inorganic-rich solid-electrolyte interphase enables 91.9% initial Coulombic efficiency and 96.5% capacity retention over 5,000 cycles.

**Supplementary Information:**

The online version contains supplementary material available at https://doi.org/10.1007/s40820-026-02339-w.

## Graphical abstract

**Figure:**
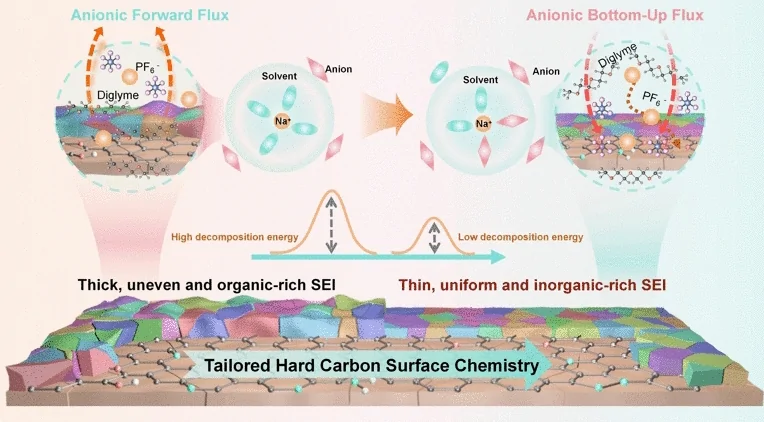

## Introduction

The escalating demand for sustainable energy storage has positioned sodium-ion batteries (SIBs) as a compelling alternative to lithium-ion batteries, owing to sodium’s natural abundance, inherent safety, and manufacturing compatibility [1]. Hard carbon (HC) is the most commercially viable anode for SIBs due to its high theoretical capacity (> 300 mAh g^−1^), suitable redox potential (~ 0.1 V vs. Na^+^/Na), and low cost [2, 3]. However, its practical application is severely hindered by low initial Coulombic efficiency (ICE) and inadequate cycling stability [4, 5]. These drawbacks originate from the unstable solid-electrolyte interphase (SEI), whose uncontrolled formation continuously consumes sodium and degrades the electrode [6, 7]. The structure, composition, and properties of the SEI directly dictate the kinetics of ion transport, the extent of irreversible sodium consumption, and the long-term structural integrity of the electrode.

Extensive research has focused on engineering the SEI to enhance battery performance. One approach center on electrolyte engineering, including the use of film-forming additives and the development of concentrated electrolytes. Additives such as fluoroethylene carbonate (FEC) can preferentially reduce to form a more robust SEI, while concentrated electrolytes alter the solvation structure, shifting the primary decomposition from solvent molecules to anions [8, 9]. These strategies primarily modulate the composition of species arriving at the electrode surface, determining whether solvents or anions dominate the flux approaching the interface. Once these species reach the surface, their subsequent decomposition pathways are largely governed by the chemical nature of the electrode. The HC surface, with its inherent defects and random functional groups, remains a reactive participant in these interfacial reactions [10].

In parallel, efforts to modify the HC anode have focused on two main directions, including bulk structural engineering and surface functionalization. Bulk modifications, such as heteroatom doping and closed‑pore construction, enhance Na^+^ storage capacity but often lack precise control over interfacial processes [11, 12]. In contrast, surface functionalization offers a more targeted approach directly to regulate interfacial chemistry and to tailor its surface properties. For instance, implanting carbonyl (C = O) groups can catalyze anion decomposition [13, 14], while grafting passivation layers converts reactive surface groups into more stable moieties [15]. Despite these advances, most surface treatments still regard the electrode as a static substrate that passively responds to species arriving from the electrolyte, rather than actively orchestrating the decomposition pathway.

The interplay between electrolyte composition and surface chemistry ultimately determines SEI properties. While electrolyte engineering can enrich anions at the interface, the subsequent decomposition selectivity depends critically on how the electrode surface interacts with these anions relative to solvent molecules. Our group has previously established fundamental structure–performance relationships of hard carbon and explored electrolyte engineering strategies to modulate interfacial chemistry [16, 17]. However, the extent to which the electrode surface itself can be designed to actively steer decomposition pathways, working in concert with the electrolyte, has yet to be established.

Recent studies suggest that surface chemistry can regulate interfacial ion behavior. For example, constructing a weakly solvating interface enriched with sp^2^-carbon reduces solvent adsorption and promotes anion ingress into the Na⁺ solvation sheath [18]; a polymer‑induced SEI using a polyethylene‑sulfonyl fluoride coating enriches PF_6_^−^ anions at the HC interface [19]; a targeted functionalization that converts surface oxygen groups into a Si‑O‑Si molecular layer enhances anion anchoring [20]; and a grafting technique that attaches a highly fluorinated molecule to HC assists in forming a NaF‑rich SEI [21]. These pioneering works confirm that tailored surface chemistry can positively influence SEI formation and improve electrochemical performance. Nevertheless, most existing strategies focus on introducing single functional groups or passive coating layers, which primarily serve to mitigate side reactions or enrich anions at the interface. The concept of using two distinct functional groups with complementary roles, one that strongly captures anions and another that repels solvents, to actively program a sustained, directional anionic flux from the bulk electrolyte to the interface has not been the central focus of previous studies.

**Fig. 1:**
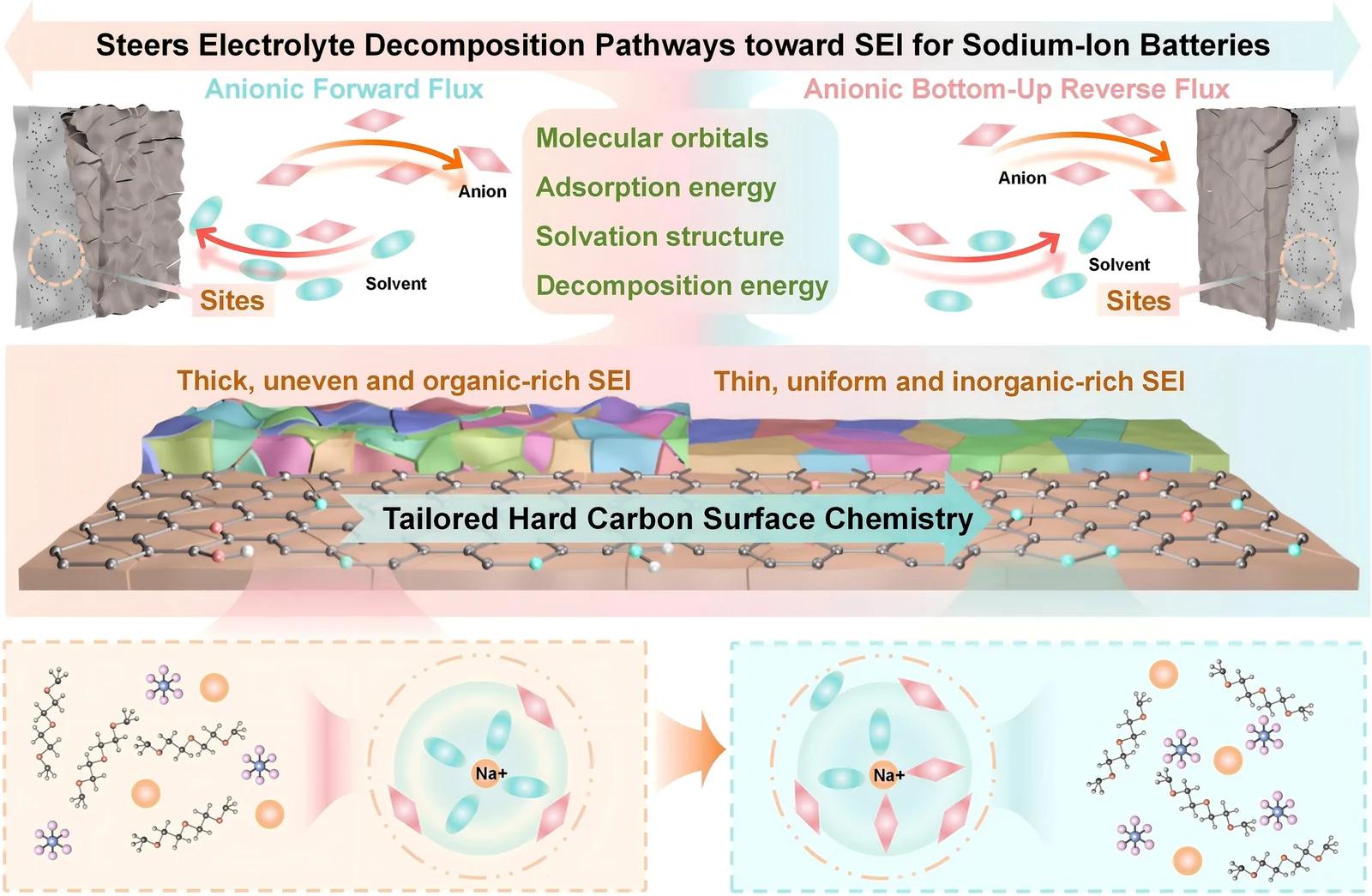
Anionic bottom-up reverse flux for steering interfacial solvation structure by molecular-level surface design. (The diamond represents the hexafluorophosphate anion, and the ellipse represents the G2 solvent molecule)

In this work, we demonstrate that molecular-level engineering of the HC surface can fundamentally redirect the competitive adsorption and decomposition pathway at the electrode–electrolyte interface. By positioning pyridinic-N and carbonyl groups as a cooperative pair, we create a precisely tailored microenvironment where the two functionalities assume distinct yet synergistic roles. Pyridinic-N acts as a Lewis base to selectively adsorb PF_6_^−^ anions, while the proximal carbonyl group simultaneously weakens solvent affinity. This functional differentiation alters the interfacial solvation structure and establishes a sustained bottom-up anionic reverse flux from the electrolyte bulk (Fig. 1). The synergistic pair enriches anions at the interface and lowers the PF_6_^−^ decomposition barrier, steering decomposition toward an anion-preferential pathway and yielding a thin, inorganic-rich SEI. The optimized anode achieves a high ICE of 91.9%, remarkable rate capability (162.1 mAh g^−1^ at 10 A g^−1^), and exceptional cycling stability with 96.5% capacity retention after 5,000 cycles. By integrating theoretical and experimental analyses, this work decouples the surface-mediated anionic dynamics and provides a rational design pathway for active interphase engineering in advanced batteries.

## Experimental Section

### Materials

In this study, peanut shells (PS) were chosen as the precursor for hard carbon synthesis. Prior to heat treatment, the PS undergo crushing in a crusher and are then passed through a 200-mesh sieve. The resulting powder is subsequently soaked in 0.1 M dilute hydrochloric acid for 24 h, thoroughly washed with ultrapure water, and finally dried. The obtained PS was heated to 350 °C at a rate of 5 °C min^−1^ and kept for 3 h under an air atmosphere. The obtained precursor is termed as pre-PS. Subsequently, pre-PS was heated to 1400 °C at a rate of 2 °C min^−1^ and kept for 3 h in a tube furnace under a flowing argon atmosphere. After cooling to room temperature, the target hard carbon (termed P–HC) was obtained. Additionally, P–HC was heated to 600 °C at a rate of 5 °C min^−1^ and kept for 0.5 h under a flowing ammonia atmosphere, and the obtained sample was denoted as R–HC.

### Electrochemical Measurement

Electrochemical measurements were conducted using a CR2032 coin-type cell. The working electrode was prepared by coating a slurry of the active material, super P, and CMC at a weight ratio of 8: 1: 1 on copper foil followed by drying at 80 °C overnight. The mass loading of the active material is 1.0–1.2 mg cm^−2^. A pure Na sheet was used as the counter electrode, and glass fiber was used as a separator. The applied electrolyte was 1 M NaPF_6_ dissolved in diglyme (G2). The cells were assembled in an argon-filled glove box with moisture and oxygen concentrations below 1 ppm. The galvanostatic charge/discharge cycles of the cells were measured between 0.01 and 3.0 V versus Na/Na^+^ at various current densities using a LAND battery testing instrument under 27 °C. Cyclic voltammograms (CV) were recorded at a scan rate of 0.1 mV s^−1^ in a potential range of 0.01–3.0 V. Electrochemical impedance spectra (EIS) were recorded at an amplitude of 5.0 mV for a frequency range of 100 kHz to 0.01 Hz. Both the CV and EIS measurements were carried out on a CHI 660C electrochemical workstation. The pouch cell was assembled by using Na_4_Fe_3_(PO_4_)_3_P_2_O_7_ (NFPP) as cathode, the mass loading of anode is 1.4 mg cm^−2^, the mass loading of cathode is 4.1 mg cm^−2^, and the N/P ratio was 1.09. The applied electrolyte was 1 M NaPF_6_ dissolved in diglyme (G2). The electrochemical performance of pouch cell was measured between 1.5 and 3.4 V.

## Results and Discussion

### Theoretical Insights into Anionic Dynamics on HC Surfaces

The formation of the SEI is intrinsically governed by the competitive adsorption between solvent molecules and anions at the electrode–electrolyte interface, which dictates the primary decomposition pathway [9]. While heteroatom doping alters carbon surface properties, how specific functional groups atomically direct these interfacial reactions remains unclear. Previous studies often conflate individual contributions of N or O sites [22], leaving their synergy in steering interfacial dynamics unexplored.

**Fig. 2:**
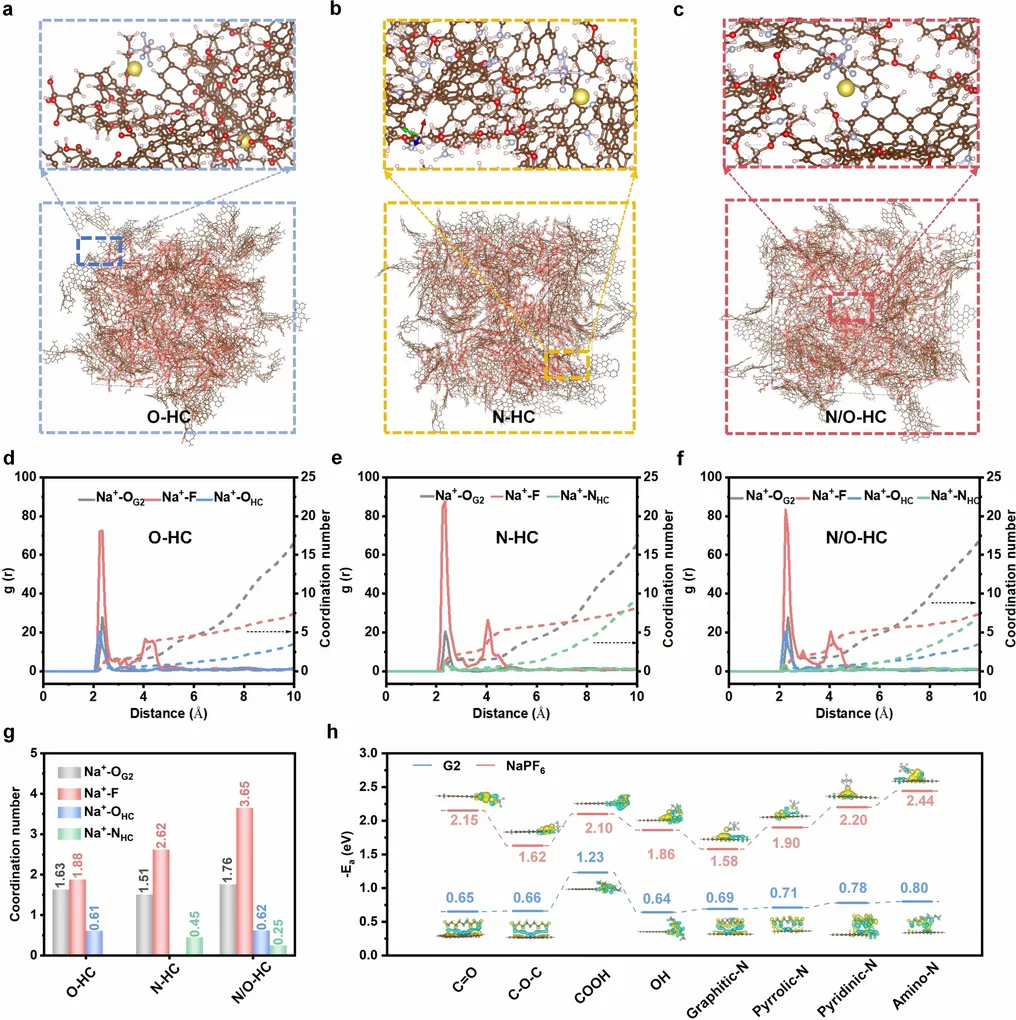
**a–c** Snapshots of the structure of O–HC, N–HC, and N/O–HC immersed in the electrolyte calculated from MD simulations, and the atoms are represented by *C*: brown, *O*: red, *H*: pink, *P*: purple, and *F*: blue. **d–f** Na^+^ radial distribution function obtained from MD simulation. **g** RDF values. **h** G2 and PF_6_^−^ adsorption energy

To investigate the effect of the functionalized HC surface on SEI formation, multiscale simulations were performed. Crucially, the molecular dynamics (MD) simulations explicitly account for the modulation of the solvation structure by the functionalized carbon surface, moving beyond the description of isolated electrolyte behavior with ideal HC surface. As illustrated in Fig. 2a-c, three distinct HC models, including oxygen-functionalized (O–HC), nitrogen-functionalized (N–HC), and nitrogen–oxygen co-functionalized (N/O–HC), were immersed in a 1 M NaPF_6_-diglyme (G2) electrolyte. This setup captures the heterogeneous interfacial environment, where solvation structures are perturbed near the surface, leading to localized anion enrichment, altered desolvation processes, and potential co-intercalation behavior, in contrast to the unperturbed state in bulk-like regions.

Radial distribution functions (RDFs) derived from the MD simulations reveal a spatially reorganized distribution of species from the bulk to the interface (Fig. 2d-f). As a baseline, the pristine electrolyte (without any carbon surface) exhibits a solvent-dominated solvation sheath, with Na^+^–F and Na^+^–O(G2) coordination numbers of 1.62 and 2.76, respectively (Fig. S1). Upon introducing functionalized surfaces, quantitative analysis of coordination numbers (Fig. 2g) demonstrates that Na^+^–F(PF_6_^−^) coordination increases substantially in the N/O–HC system (3.65) versus O–HC (1.88) and N–HC (2.62), while Na^+^–O(G2) coordination remains stable. Population analysis (Tables S1-S3) further confirms solvation structures containing two PF_6_^−^ anions are significantly enriched on N/O–HC (11.53% vs. 3.34% for O–HC). These observations establish a thermodynamically favorable concentration gradient of anions from the electrolyte bulk toward the N/O-functionalized interface, which provides the driving force for a sustained directional transport of anions, a process referred to as a bottom-up anionic flux.

Furthermore, as shown in density functional theory (DFT) calculations results (Figs. 2h and S1-S2). All functional groups exhibit a stronger affinity for NaPF_6_ than for the G2 solvent, with amino-N (−2.44 eV) and pyridinic-N (−2.20 eV) showing exceptional anion adsorption. This establishes a clear thermodynamic driving force for anion enrichment at the interface, where these N sites act as Lewis bases coordinating with the P atom in PF_6_^−^ [23]. The thermodynamic preference, combined with the spatial proximity revealed through MD simulations, establishes the essential conditions for sustaining a directional anionic flux. In this process, anions are continuously drawn from the bulk by the string adsorption sites at the interface, maintaining a steady-state concentration gradient. A comparative analysis of spatial configurations (Fig. S3) reveals critical fine-tuning. In the pyrrolic-N/C = O pair, both sites show comparable PF_6_^−^ adsorption (−1.90 and −2.14 eV), indicating competitive co-adsorption. In contrast, the pyridinic-N/C = O pair exhibits distinct functional differentiation, in which pyridinic-N serves as the primary anchor for PF_6_^−^ (−2.28 vs. −1.82 eV for C = O), while the C = O group shows the weakest solvent interaction (−0.47 eV). This creates a synergistic interface where pyridinic-N dynamically captures incoming anions from the bulk, while C = O sustains a solvent-depleted local environment that enables unimpeded anion transport toward the surface, effectively orchestrating a continuous bottom-up flux [24].

Molecular orbital analysis provides deeper insight into the interfacial electronic interactions. The pyrrolic‑N/NaPF_6_ complex exhibits a significantly stabilized LUMO (−3.75 vs. −2.86 eV for pristine NaPF_6_), while the pyridinic‑N/NaPF_6_ complex shows a slightly higher LUMO (−2.79 eV) but a substantially lowered HOMO (−5.28 eV compared to −5.11 eV for the pyrrolic‑N complex and −4.96 eV for pristine NaPF_6_, Figs. S5-S7). Although the LUMO of the pyridinic‑N/NaPF_6_ complex is not lower than that of pristine NaPF_6_, the strong adsorption affinity of pyridinic‑N for PF_6_^−^ (−2.28 eV) can locally enrich anions at the interface and alter the Na⁺/PF_6_^−^ ion‑pair configuration, thereby reducing the kinetic barrier for PF_6_^−^ decomposition. The complementary roles of orbital energy shifts and interfacial adsorption collectively steer the decomposition toward an anion‑preferential pathway [8]. Beyond steering decomposition, N–O co-functionalization optimally modulates Na^+^ adsorption, critically enhancing storage reversibility. Analysis of isolated functional groups shows strong Na^+^ adsorption for pyrrolic-N (−3.25 eV) and pyridinic-N (−2.54 eV) (Fig. S8). However, coupling these N sites with C = O groups significantly moderate the adsorption strength (Fig. S9). In the pyrrolic-N/C = O configuration, the energy decreases to −2.40 eV. For pyridinic-N/C = O, both sites exhibit identical, moderated energies of −2.59 eV. This systematic ensures sufficient Na^+^ binding for efficient storage while promoting facile desorption for highly reversible cycling, thereby mitigating irreversible sodium trapping.

### Material Synthesis and Structural Characterization

**Fig. 3:**
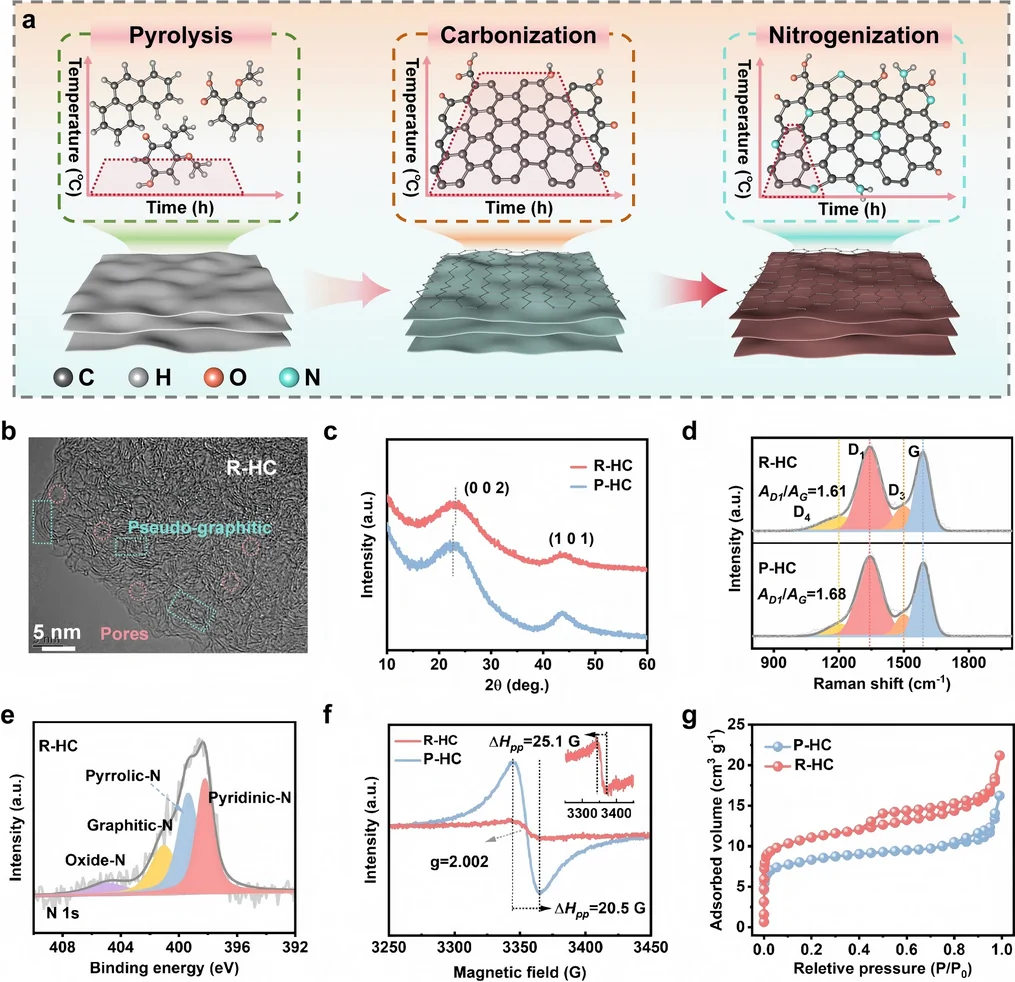
**a** Schematic of evolution process of biomass-derived carbon structure. **b** HRTEM of R–HC. **c** XRD patterns. **d** Raman spectra. **e** High-resolution N 1*s* spectrum. **f** EPR plots. **g** N_2_ adsorption–desorption isotherms

Guided by theoretical predictions, a scalable in situ structuring strategy to synthesize N–O co-functionalized HC was developed. As illustrated in Fig. 3a, peanut shell-derived HC (P–HC) was prepared through acid pickling, pre-oxidation, and high-temperature carbonization. R–HC was obtained by further treating P–HC in an ammonia atmosphere. Fourier transform infrared (FTIR) spectra of the pristine (PS) and pre-oxidized peanut shell (pre-PS) (Fig. S10a) confirm the evolution of surface functionalities during pre-oxidation. Both samples show aromatic C–H bending (800–900 cm^−1^), C = C skeletal vibrations (1512–1620 cm^−1^), and O–H stretching (~ 3425 cm^−1^) [16]. Notably, the intensities of the C–O–C and C–O stretching modes (1000–1350 cm^−1^), prominent in PS, are substantially weakened in pre-PS, indicating sacrificial decomposition of oxygen-containing groups and the formation of a thermally stable, cross-linked framework dominated by C–C bonds [25]. This structural evolution suppresses graphitic ordering during carbonization and promotes a disordered carbon matrix. Thermogravimetric analysis in air (Fig. S10b) reveals that PS undergoes a distinct two-stage mass loss, involving decomposition of hemicellulose/cellulose (commencing at ~ 260 °C) followed by lignin decomposition (~ 375 °C) [16, 26]. These further underscore the formation of a stable cross-linked structure after pre-oxidation, essential for preserving structural integrity throughout carbonization.

The microstructures of P–HC and R–HC were characterized by transmission electron microscopy (TEM). Both samples display a lamellar morphology (Fig. S11), with high-resolution TEM (HRTEM) revealing distinct structural evolution after NH_3_ treatment (Fig. 3b). P–HC consists of chaotically stacked short-range graphene-like layers with an interlayer spacing of 0.393 nm (Fig. S12a-c). In contrast, R–HC exhibits more ordered and extended graphitic domains with a reduced interlayer spacing of 0.388 nm (Fig. S12d-f). Abundant nanovoids, marked by circles, are observed in both materials and are expected to contribute to sodium storage. XRD patterns (Fig. 3c) and fitting results (Fig. S13) further confirm the structural differences of P–HC and R–HC, consistent with HRTEM results. The increase in the in-plane crystallite size (*L*_a_) from 4.486 nm (P–HC) to 5.303 nm (R–HC) indicates lateral growth of graphitic domains and reduced in-plane defects [27]. The slight decrease in the stacking height (*L*_c_) from 1.197 to 1.079 nm may result from the etching of amorphous carbon regions by NH_3_, which preferentially removes disordered carbon while refining the crystalline domains. More detailed crystallinity information emerged from the Raman spectra (Fig. 3d), revealing two broad bands at ~ 1350 and ~ 1590 cm^−1^ for D1 and G, corresponding to the defect and crystalline graphite carbon microstructure, respectively [12, 28, 29]. The *A*_D1_/*A*_G_ of R–HC (1.62) was smaller than that of HC (1.68), indicating a more ordered graphite structure and fewer defect sites in R–HC.

X-ray photoelectron spectroscopy (XPS) analysis reveals significant evolution in surface chemistry (Fig. S14). The nitrogen content increases substantially from 0.99% in P–HC to 2.78% in R–HC, confirming effective nitrogen incorporation. Deconvolution of the C 1*s* spectra (Fig. S15a, d) shows an increased intensity of the C–N/C–O peak (286.95 eV) in R–HC, confirming N-incorporation [30, 31]. The O 1*s* spectra (Fig. S15b, e) reveal a transformation of oxygen species, with a decrease in C–O/C = O groups and an increase in the component at 533.3 eV [32, 33]. Most critically, the N 1*s* spectrum of R–HC (Figs. 3e and S15c, f) confirms the presence of pyridinic-N (398.6 eV, 34.3%), pyrrolic-N (399.74 eV, 37.1%), graphitic-N (401.42 eV, 21.8%), and oxidized-N (405.18 eV, 6.8%), while P–HC shows negligible nitrogen [34]. This synergistic nitrogen configuration, combined with the modulated oxygen and carbon bonding environments, is expected to enhance the electrochemical performance of R–HC.

FTIR spectra (Fig. S16) illustrate the chemical evolution induced by NH_3_ treatment. The band centered at 1615 and 1580 cm^−1^ may be attributed to the residual C = O and –NH_2_ group [35, 36]. Notably, the intensity of the peak at 1451 cm^−1^ (aliphatic C–H bending) attenuates in R–HC, indicating removal of defective hydrogen-terminated carbon. Furthermore, the characteristic aromatic C–H out-of-plane bending peak at 880 cm^−1^, clearly visible in P–HC, disappears completely in R–HC, suggesting substitution of edge C–H groups by nitrogen species during NH_3_ treatment.

Electron paramagnetic resonance (EPR) measurements (Fig. 3f) further demonstrate the electronic structure modification. The drastically reduced signal intensity in R–HC confirms passivation of paramagnetic defects, while the broadened linewidth (20.5 to 25.1 G) reflects enhanced spin–orbit coupling due to nitrogen incorporation, indicating transition to a delocalized *π*-electron system favorable for charge transport [22, 37]. N_2_ physisorption (Fig. 3g) reveals a microporous structure in both materials, with R–HC exhibiting higher specific surface area (111.27 vs. 68.93 m^2^ g^−1^) and pore volume (0.060 vs. 0.041 cm^3^ g^−1^). Pore size distribution (Fig. S17) analysis shows developed micropores (0.5–1 nm) and mesopores (4.1–5.5 nm) in R–HC. The resulting hierarchical pore structure in R–HC is expected to facilitate ion accessibility and transport within the electrode [27]. Small-angle X-ray scattering (SAXS) measurements were further performed to probe the closed pore structure (Fig. S18). The SAXS profiles of P–HC and R–HC exhibit nearly identical scattering intensity trends across the measured q range, with only a marginally higher shoulder in the low q region for R–HC. This indicates that the volume fraction and size distribution of closed pores in R–HC are only slightly increased compared to P–HC, suggesting that the NH_3_ treatment does not fundamentally alter the bulk closed pore architecture.

### Electrochemical Performance and Sodium Storage Kinetics

**Fig. 4:**
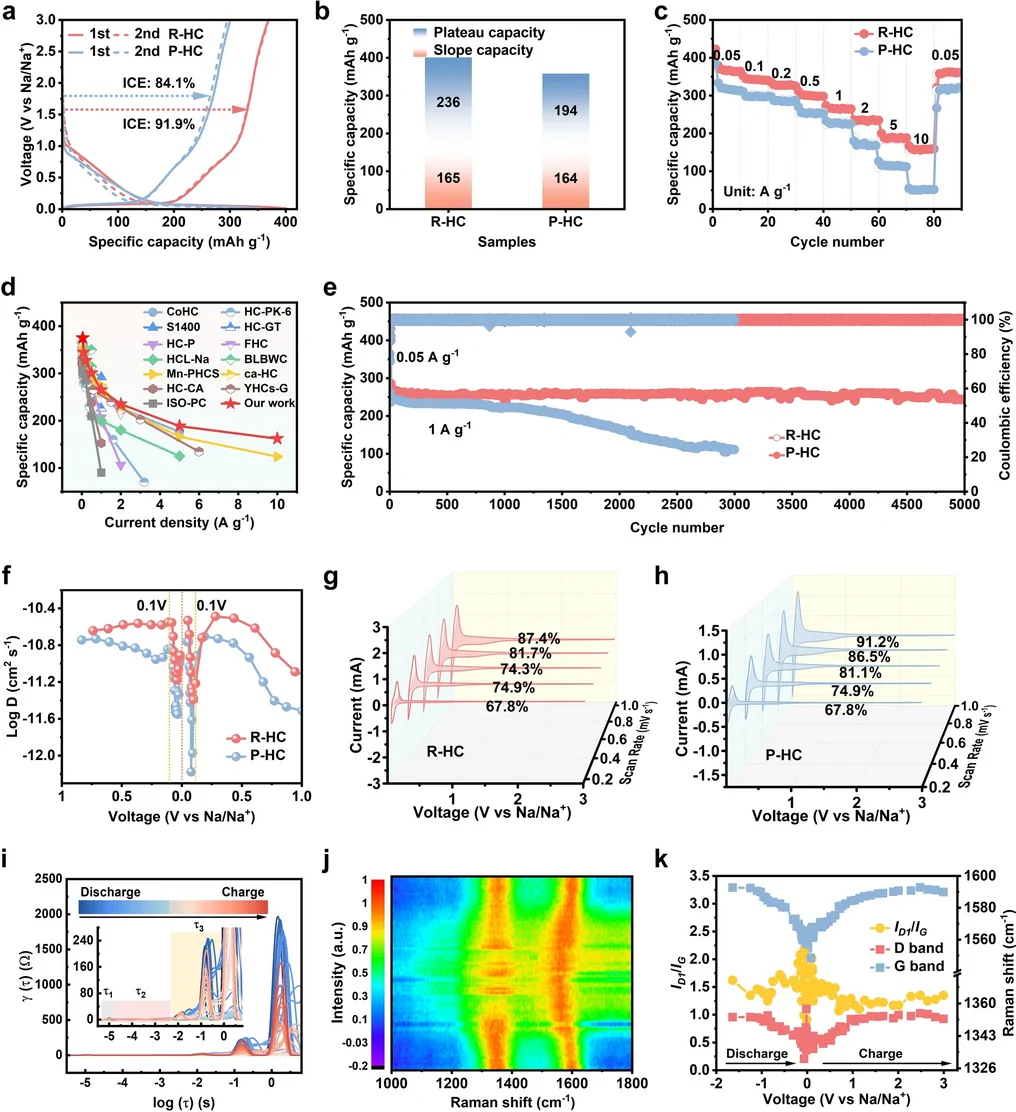
**a** GCD plots at 0.05 A g^−1^. **b** Comparison of the plateau and sloping capacity of electrodes based on the 1st cycle. **c** Rate performance. **d** Rate comparison of R–HC with other reported HC anode. **e** Cycling performance at 1 A g^−1^. **f** GITT curves. **g**, **h** The diffusion and capacitive contribution at various scan rates for R–HC and P–HC. **i** DRT plots of R–HC derived from in situ EIS data collected during the first cycle. **j** In situ Raman spectra of R–HC during discharging and charging process. **k** Corresponding Raman shift of D1 band, G band, and *I*_D1_*/I*_G_ values

The electrochemical performance of all samples was tested in half-cells with metallic Na as counter electrodes. The R–HC electrode demonstrates substantially higher current response, indicating enhanced reaction kinetics. Galvanostatic charge–discharge (GCD) profiles at 0.05 A g^−1^ (Fig. 4a) show R–HC delivers 368.2 mAh g^−1^ with 91.9% ICE, surpassing P-HC (301.1 mAh g^−1^, 84.1% ICE). Three parallel R–HC electrodes tested under the same conditions yield ICE values of 91.6%, 90.2%, and 91.8% (Fig. S19), confirming good reproducibility. It is worth noting that the NH_3_ treatment simultaneously induces bulk structural evolution and surface N–O co-functionalization. The bulk changes primarily contribute to the enhanced plateau capacity by providing additional sodium storage sites, whereas the surface chemistry governs the high ICE and long-term cycling stability by steering the formation of an inorganic-rich SEI. Capacity distribution analysis indicates the performance gain primarily originates from the plateau region (Fig. 4b, 236 vs. 194 mAh g^−1^ for R–HC and P–HC, respectively), while the sloping capacities remain similar. In the second cycle (Fig. S20), the sloping and plateau capacities decrease to 144/228 mAh g^−1^ for R–HC and 126/177 mAh g^−1^ for P–HC. This attenuation is attributed to irreversible consumption of active sodium and electrolyte during initial SEI formation. The smaller capacity loss in R–HC, particularly in the plateau region, underscores the role of the stable, inorganic-dominated SEI in mitigating continuous side reactions and preserving reversible storage sites. In contrast, P–HC experiences more severe capacity fade due to its thicker, organic-rich SEI and higher defect density.

Cyclic voltammetry (CV) at 0.1 mV s^−1^ (Fig. S21) reveals redox peaks near 0.1/0.01 V, corresponding to the low-voltage plateau, along with a broad SEI formation peak around 0.67 V [38]. The rate capability of R–HC significantly surpasses that of P–HC, maintaining 162.1 mAh g^−1^ at 10 A g^−1^ compared to 54.5 mAh g^−1^ for P–HC (Fig. 4c). Analysis of the GCD profiles (Fig. S22) reveals that capacity attenuation at high rates primarily originates from the plateau region, suggesting kinetic limitations in pore-filling processes under substantial polarization. The performance of R–HC exceeds most reported plateau-type hard carbons (Fig. 4d and Table S4) [21, 26, 39–49]. Long-term cycling at 1 A g^−1^ demonstrates exceptional stability for R–HC, with 96.5% capacity retention after 5,000 cycles and near-perfect Coulombic efficiency (Fig. 4e). In contrast, P–HC suffers rapid degradation, declining from 224 to 107 mAh g^−1^ after 3000 cycles.

The sodium storage kinetics were systematically investigated through a suite of electrochemical techniques. Electrochemical impedance spectra (EIS, Fig. S23) demonstrate significantly lower overall impedance for R–HC both before and after 100 cycling, with reduced charge-transfer resistance (*R*_ct_) and diffusion impedance (*Z*_w_), indicating enhanced interfacial charge transfer and ion diffusion kinetics [50]. Complementing the EIS results, galvanostatic intermittent titration technique (GITT) measurements performed at a pulse current of 0.05 A g^−1^ (Fig. S24) provide further insight into Na^+^ diffusion behavior. The diffusion coefficient (*D*_Na+_), calculated during sodiation/desodiation (Fig. 4f), remains relatively stable in the high-potential slope region, consistent with rapid adsorption processes on the carbon surface. A pronounced drop in *D*_Na+_ occurs in the low-potential plateau region, attributed to Na^+^ intercalation into the carbon interlayers, followed by a characteristic rebound near 0.05 V associated with pore-filling processes [51, 52]. To decouple the capacitive and diffusion-controlled contributions to the total storage capacity, CV test was performed at various scan rates (Fig. S25a, c). The peak current of R–HC increases more markedly with scan rate compared to P–HC. The *b* values obtained from the log(*i*)–log(*v*) relationship (Fig. S25b, d) are lower for R–HC (0.69 and 0.63) for the two redox peaks) than for P–HC (0.78 and 0.69) [53], coupled with quantitative analysis of the diffusion contributions (Fig. 4g, h), indicating a greater contribution from diffusion-controlled processes and faster reaction kinetics in R–HC.

In situ EIS coupled with distribution of relaxation times (DRT) analysis was employed to dynamically monitor the evolution of the SEI and its influence on electrochemical kinetics during the first cycle. The continuous contraction of the high-frequency semicircle indicates the progressive formation and stabilization of a highly ion-conductive SEI. The evolution of the mid-frequency semicircle, corresponding to the *R*_ct_, experiences increase during the initial Na^+^ adsorption/intercalation and decreases upon nanopore filling, due to the formation of efficient conduction pathways. In Fig. S26a, R–HC exhibits a steeper low-frequency slope and more stable impedance response throughout cycling than that in P–HC (Fig. S26b), indicating enhanced Na^+^ diffusion and interfacial stability attributable to its robust SEI. DRT deconvolution in Fig. 4i resolves three distinct relaxation processes, including *τ*_1_ (contact resistance), *τ*_2_ (Na^+^ transport through the SEI, *R*_sei_), and *τ*_3_ (*R*_ct_) [22]. The sequential right-shift and subsequent left-shift of the *τ*_2_ peak suggest dynamic SEI reorganization toward a more conductive architecture. Notably, R–HC demonstrates significantly lower intensity across all τ peaks compared to P–HC (Fig. S27), quantitatively confirming its superior kinetics.

The structural evolution of R–HC during the first cycle has been investigated using in situ Raman spectroscopy (Fig. 4j). As shown in Fig. 4k, a continuous redshift of both the D1 and G bands is observed during discharge, accompanied by a blueshift back to the original positions upon charge, demonstrating highly reversible lattice expansion and electron transfer processes associated with sodium (de)intercalation. This phonon softening (redshift) originates from the electron donation from sodium to the carbon matrix, populating the π* antibonding orbitals and weakening the C–C bonds, coupled with mechanical strain induced by ion insertion [54]. The evolution of the *I*_D1_/*I*_G_ ratio further delineates the stage-specific sodium storage behavior. Generally, the initial decrease above 1 V suggests sodium adsorption at defect sites, partially passivating these regions; the subsequent increase between 1.0 and 0.5 V reflects intensified intercalation into graphitic layers, generating new defects and strain; the second decrease from 0.5 to 0.1 V implies a saturation of intercalation and the onset of pore filling, creating a more ordered local environment; and the final sharp increase below 0.1 V signifies extensive nanopore filling and quasi-metallic sodium deposition, introducing significant lattice distortion and defect generation. During charging, the ratio decreases as sodium is extracted from the pores, relieving internal stress, followed by a gradual increase and stabilization at a slightly elevated value, indicative of minor irreversible defect formation and SEI contribution.

Ex situ XRD patterns of R–HC at different voltages (Fig. S28) show that during the discharging process from 1 to 0.1 V, there is no shift in the (002) peak, indicating that Na^+^ is mainly adsorbed on the carbon surface in the slope region. Further discharging to 0.01 V causes a significant leftward shift of the (002) peak, corresponding to the sodium filling within the nanopores in the plateau region. The storage of sodium clusters in anode was further confirmed by the discoloration of phenolphthalein. R–HC electrode was discharged to different cutoff voltages, and soaked in the phenolphthalein solution. The digital image in Fig. S29 displays the discoloration of the phenolic solution. At 0.1 V, minimal color change is observed, indicating that the slope capacity of R–HC primarily resulted from ionic adsorption. As discharge progressed, the discoloration intensified, with R–HC exhibiting a high presence of sodium clusters at 0.01 V. All these results demonstrate that sodium ions primarily undergo adsorption in the slope region, intercalation into the carbon layers, and further reduction to sodium clusters filling in enclosed pores in the plateau region.

### Interfacial Analysis of the Inorganic-Rich SEI

Theoretical calculations of PF_6_^−^ decomposition energy barriers provide fundamental insight into the interfacial reactions guiding SEI formation (Fig. S30). The dissociation energy for PF_6_^−^ is calculated to be 2.93 eV on the oxygen-rich surface and increases to 3.31 eV on the nitrogen-rich surface. Strikingly, this energy barrier plummets to 0.77 eV on the N–O co-functionalized surface. This drastically reduced energy requirement unequivocally predicts a catalytically enhanced and preferential decomposition of PF_6_^−^ anions on the R–HC anode, steering the formation of an inorganic-rich SEI.

To experimentally track the decomposition process, ex situ XPS measurements were performed on R–HC and P–HC electrodes discharged to different potentials (0.6, 0.3, and 0.01 V) during the first sodiation (Fig. S31). The F 1*s* spectra reveal a clear difference in the onset potential of PF_6_^−^ reduction. On R–HC, a distinct NaF peak (683.2 eV) already appears at 0.6 V and its intensity increases progressively with deeper discharge. In contrast, on P–HC, only the P–F peak from undecomposed PF_6_^−^ (686.6 eV) is observed at 0.6 V [45], a weak NaF signal emerges only at 0.3 V and becomes noticeable at 0.01 V. This potential-dependent evolution directly demonstrates that the N/O-functionalized surface promotes PF_6_^−^ reduction at a significantly higher potential, i.e., an anion-preferential decomposition pathway, whereas on the P–HC surface, solvent decomposition dominates until lower potentials.

**Fig. 5:**
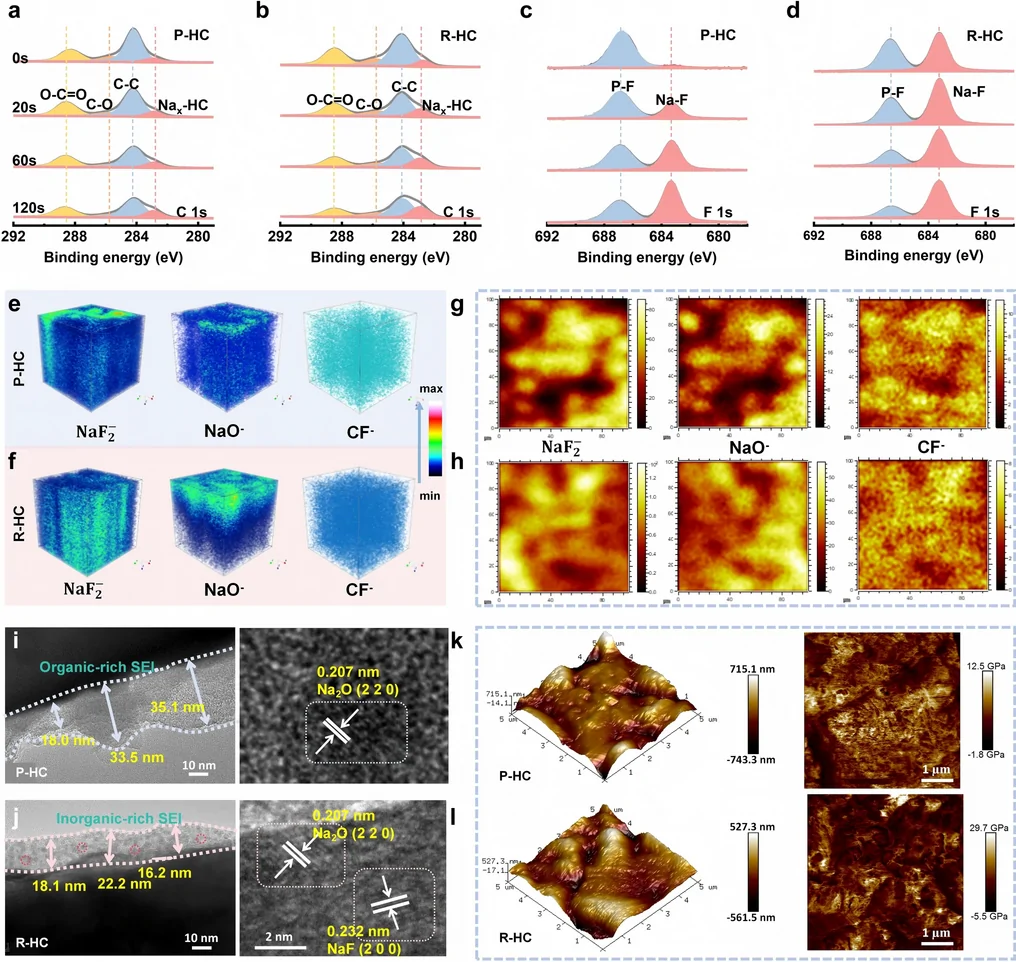
**a**, **b** Depth-profiling XPS spectra of C 1*s* spectra of P–HC, and R–HC. **c**, **d** Depth-profiling XPS spectra of F 1*s* spectra of P–HC, and R–HC. **e**, **f** 3D TOF–SIMS visual maps showing the distribution of inorganic and organic species in the SEI of P–HC and R–HC. **g**, **h** Surface species distributions of P–HC and R–HC. **i**, **j** HRTEM images of P–HC and R–HC after cycles. **k**, **l** Modulus distribution of P–HC surface and R–HC surface

Subsequently, XPS depth profiling with Ar^+^ etching was employed to experimentally elucidate the composition and structure of the SEI. Comparative analysis between the R–HC and P–HC samples reveals marked differences in SEI evolution, confirming the theoretical prediction. For the C 1*s* spectrum and fitting results (Figs. 5a, b and S32a), the peak at 282.9 eV (assigned to carbides or carbon in a strongly reduced environment) increases significantly with etching depth in both samples, indicating exposure of the underlying carbon substrate [55]. The peaks corresponding to organic species, C = O/O–C = O (288.5 eV) and C–O (285.6 eV), decrease with etching, suggesting the gradual removal of the organic outer SEI layer [45, 56]. The O 1*s* spectra (Fig. S33) and the calculated content ratio of functional groups (Fig. S32b) show that the component at 530.6 eV (inorganic oxides, Na_2_O) constitutes the majority in both materials and remains stable with etching. However, the more pronounced decrease in the organic oxygen peak (532.3 eV) in R–HC further supports the formation of an inorganic-rich inner SEI structure in R–HC. In the F 1*s* spectra (Fig. 5c, d), the peak at 683.2 eV (assigned to NaF) [57] increases substantially with etching time in both samples, but much more markedly in R–HC compared to P–HC. Concurrently, the P–F species peak (686.6 eV) diminishes more rapidly in R–HC (Fig. S32c) [45]. This indicates that the SEI on R–HC contains a higher proportion of NaF in the inner layer, a direct consequence of the catalytically enhanced PF_6_^−^ decomposition, which is critical for ion transport and interfacial stability, while P–F compounds are predominantly located near the surface.

To further probe the compositional distribution within the SEI, time-of-flight secondary ion mass spectrometry (TOF–SIMS) was employed for elemental visualization of the cycled electrodes. As depicted in Figs. 5e, f and S34-S35, the R–HC electrode exhibits a continuous increase in NaF_2_^−^ and NaF^−^ signals with sputtering time, indicating a progressively enriched and homogeneous distribution of NaF throughout the inner SEI. This is accompanied by a stronger NaO^−^ signal, suggesting co-formation of sodium oxides. In contrast, P–HC shows markedly weaker intensities for both NaF^−^ and NaO-related fragments, reflecting its inferior inorganic content. Notably, the PF_6_^−^ signal in R–HC remains low and predominantly localized near the surface, consistent with efficient and preferential decomposition of PF_6_^−^ anions catalyzed by the functionalized surface. Moreover, significantly attenuated CF^−^ and C_2_H_3_O^−^ signals in R–HC confirm effective suppression of organic solvent and binder decomposition [8]. These findings collectively affirm that the tailored surface chemistry of R–HC directs electrolyte decomposition toward the formation of a compact, inorganic-rich SEI with minimal organic by-products [45]. Two-dimensional overlay mapping images further demonstrate that the proportion of inorganic components higher than that in P–HC (Figs. 5g, h and S36).

These compositional differences are directly visualized by HRTEM (Fig. 5i, j), which shows a thick and inhomogeneous SEI on P–HC, contrasted with a thin and uniform SEI on R–HC. Notably, well-crystallized Na_2_O domains are identified within the SEI of R–HC, coexisting with the abundant NaF matrix as revealed by XPS and TOF–SIMS. The presence of Na_2_O, likely derived from the reduction of surface oxygen groups, complements the NaF-rich framework by enhancing the overall mechanical integrity and ion transport properties of the SEI. This multi-component inorganic composite forms a highly ordered and ion-conductive inner layer, which is critical for interfacial stability and fast charge transfer. As revealed in the SEM images following long-term cycling at 1 A g^−1^ (Fig. S37), the R–HC electrode displays a remarkably smooth surface with minimal microcracks or sodium plating, evidencing uniform SEI formation and superior ionic connectivity. In stark contrast, the P–HC electrode suffers from significant surface deterioration. Notably, HRTEM of the R–HC electrode after cycles (Fig. S38) shows that the SEI remains thin and uniform, further confirming its long-term structural integrity. Atomic force microscopy (AFM) provides additional insight into the mechanical properties of the SEI layers. Three-dimensional topography reconstruction and nanomechanical mapping (Fig. 5k, l) reveal that R–HC possesses a markedly uniform surface with reduced height variation after cycling, characteristic of a homogeneous SEI favorable for ion transport [7]. Quantitative measurements show a higher average Young’s modulus of 7.6 GPa for R–HC, compared to 4.7 GPa for P–HC, consistent with its inorganic-rich composition. This enhanced mechanical integrity helps maintain SEI stability during cycling, mitigates particle fracturing, and limits continuous electrolyte decomposition.

### Full Cell Demonstration and Practical Viability

**Fig. 6:**
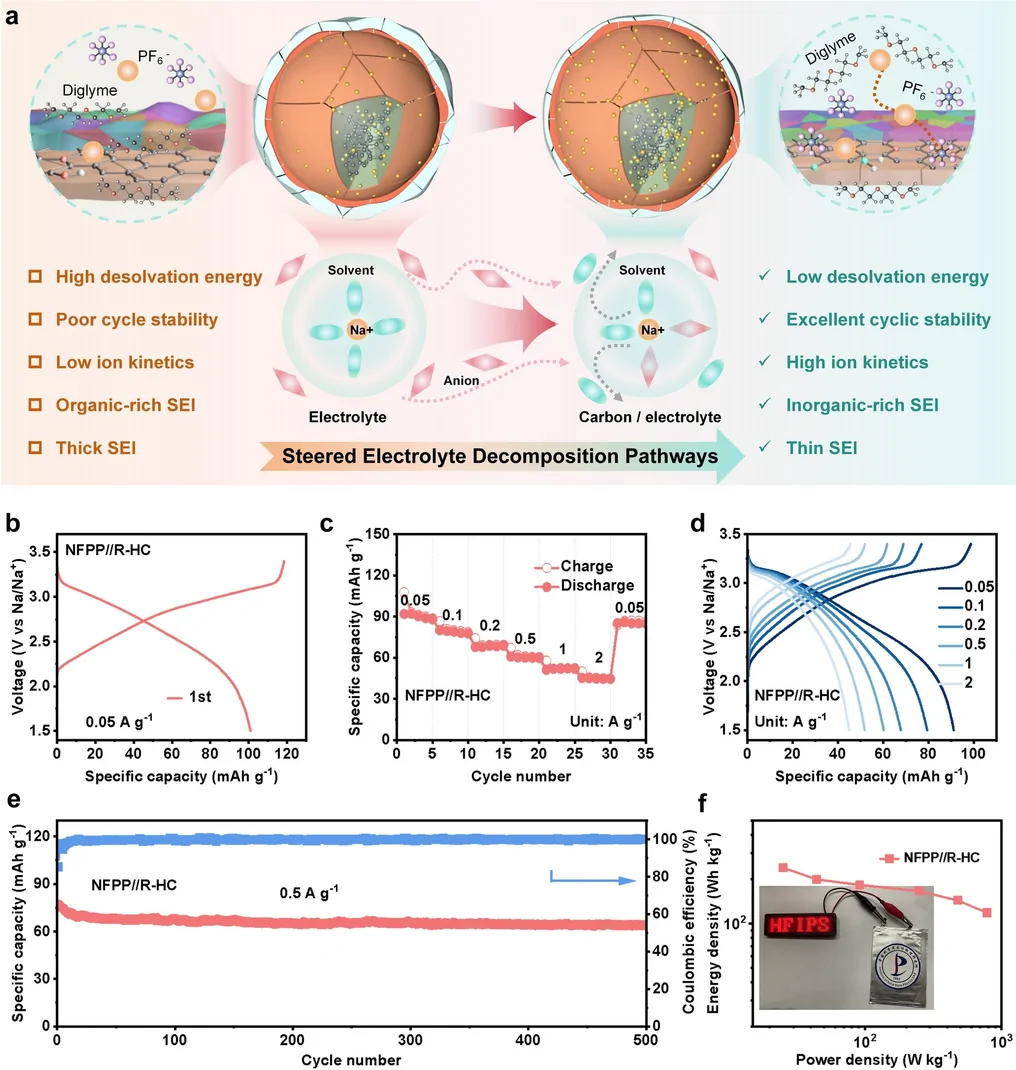
**a** Schematic illustration of SEI formation process in ether electrolyte driven by surface functional group management. **b** Initial GCD plots of full cell. **c** Rate capability of NFPP//R–HC pouch cell. **d** GCD curves of NFPP//R–HC pouch cell at different current densities. **e** Cycling performance of NFPP//R–HC pouch cell. **f** Energy density and power density

Based on the above analyses, the superior interfacial characteristics of R-HC can be attributed to the synergistic management of nitrogen and oxygen functional groups on the carbon surface (Fig. 6a), which orchestrates an efficient bottom-up anionic assembly process. The introduced nitrogen species not only enhance electronic conductivity but also serve as catalytic sites that strengthen PF_6_^−^ adsorption via tailored Lewis acid–base interactions, thereby catalytically steering its preferential decomposition into NaF. Concurrently, the residual and reconfigured oxygen functionalities contribute to passivating electrochemically active defects and modulating the local electric field, collectively weakening solvent affinity and promoting Na^+^ desolvation. This surface regulation creates a distinctive local microenvironment that selectively enriches anions while effectively repelling solvent molecules, thereby lowering the energy barrier for ion ingress and steering electrolyte decomposition toward inorganic-rich products. The engineered anode–electrolyte interface facilitates rapid ion transport, suppresses parasitic reactions, and guarantees truly exceptional structural reversibility, thereby laying the foundation for the outstanding high-rate capability, high capacity, and extraordinary long-term cyclability of R–HC anodes in SIBs.

To investigate the practical performance of the R–HC anode, a pouch cell architecture is constructed with R–HC anode paired with Na_4_Fe_3_(PO_4_)_3_P_2_O_7_ (NFPP). The electrochemical property of the NFPP is displayed in Fig. S39. Based on the specific capacities of NFPP and R–HC, the as-assembled SIBs deliver an impressive initial discharge capacity of 92.5 mAh g^−1^ (based on cathode mass) with a high ICE of 85.2% (Fig. 6b), demonstrating exceptional compatibility and minimal irreversible loss. The full cell also demonstrates remarkable rate capability, retaining a capacity of 50.2 mAh g^−1^ at an increased current rate of 2 A g^−1^ (Fig. 6c, d). Figure 6e shows the cycling performance of NFPP//R–HC at 0.5 A g^−1^, after 500 charge–discharge cycles, a capacity retention of 91.2% can be achieved, indicating robust cycling stability. Notably, the full cell achieves an energy density of 239.1 Wh kg^−1^ and power density of 785.2 W kg^−1^ relative to total electrode mass (Fig. 6f). Furthermore, the assembled pouch cell enables stable operation of a LED light board (photo inserted in Fig. 6f), demonstrating its promising prospects for practical applications.

## Conclusion

In summary, we have demonstrated that active surface chemistry design can fundamentally redirect interfacial decomposition pathways in HC anodes for SIBs. By constructing a surface with atomically pyridinic-N and carbonyl groups as cooperative sites, we create a synergistic microenvironment that enriches PF_6_^−^ anions while repelling solvents, thereby orchestrating a bottom-up anionic reverse flux and catalyzing the formation of an inorganic-rich SEI. Through a combination of MD simulations and DFT calculations, we revealed that this tailored surface significantly enhances PF_6_^−^ adsorption and reduces the energy barrier for its decomposition, thereby steering the formation of a thin, inorganic-rich SEI. Experimental validation confirmed that the optimized anode delivers an exceptional ICE of 91.9%, remarkable rate capability (162.1 mAh g^−1^ at 10  A g^−1^), and ultralong cycling stability (96.5% capacity retention after 5,000 cycles). The NFPP//R-HC pouch cell can be cycled stably for 500 cycles with 91.2% capacity retention, demonstrating the tremendous practical application potential. In conclusion, the rational design of atomic-level surface chemistry represents a fundamental strategy for active interphase engineering. This surface-mediated guidance not only resolves the interfacial instability of HC anodes but also provides a generalizable platform for developing high-performance electrodes in advanced energy storage systems.

## Supplementary Information

Below is the link to the electronic supplementary material.

## References

[1] Y. Yang, Z.F. Wang, C.C. Du, B.W. Wang, X.Y. Li et al., Decoupling the air sensitivity of Na-layered oxides. Science **385**(6710), 744–752 (2024). 10.1126/science.adm9223 39146426

[2] C. Wu, W.J. Huang, Y.H. Zhang, Q.H. Chen, L. Li et al., Revisiting the critical role of metallic ash elements in the development of hard carbon for advancing sodium-ion battery applications. eScience **5**(3), 100371 (2025). 10.1016/j.esci.2025.100371

[3] S. Gan, Y. Huang, N. Hong, Y. Zhang, B. Xiong et al., Comprehensive understanding of closed pores in hard carbon anode for high-energy sodium-ion batteries. Nano-Micro Lett. **17**(1), 325 (2025). 10.1007/s40820-025-01833-x PMC12234992 40622590

[4] M. Li, C. Sun, R. Zhang, M. Qi, Z. Wu et al., Unveiling the electrolyte and solid electrolyte interphase in sodium ion batteries: Mechanisms, progress, and perspectives. Adv. Mater. **37**(47), e10882 (2025). 10.1002/adma.202510882 41074237 PMC12651183

[5] F.J. Wang, T.Y. Zhang, T. Zhang, T.Q. He, F. Ran, Recent progress in improving rate performance of cellulose-derived carbon materials for sodium-ion batteries. Nano-Micro Lett. **16**(1), 46 (2024). 10.1007/s40820-024-01351-2 PMC10928064 38466498

[6] K. Cui, R. Hou, H. Zhou, S. Guo, Electrolyte engineering of hard carbon for sodium‐ion batteries: from mechanism analysis to design strategies. Adv. Funct. Mater. **35**(16), 2419275 (2024). 10.1002/adfm.202419275

[7] L. Chen, M. Chen, Q.F. Meng, J. Zhang, G. Feng et al., Reconstructing elmholtz plane enables robust F-rich interface for long-life and high-safe sodium-ion batteries. Angew. Chem. Int. Ed. **63**(38), 202407717 (2024). 10.1002/anie.202407717 38963683

[8] Y. Liao, H. Liu, Y. Zhang, J. Yang, H. Ji et al., A weak-fluorine-bond molecule stabilizes hard carbon anodes for practical sodium-ion batteries. ACS Nano **19**(33), 30466–30475 (2025). 10.1021/acsnano.5c10983 40817863 PMC12755197

[9] B. Song, Z. Luo, X. Liu, Y. Peng, J. Wang et al., Bridging donor ligands enable an ultrastable graphite anode for sodium-ion batteries. Angew. Chem. Int. Ed. **137**(38), e202503027 (2025). 10.1002/ange.202503027 40734348

[10] X. Feng, F. Wu, Y. Li, Y. Fu, Y. Li et al., Critical role of ultra‐microporous tunnel structure within hard carbon in boosting sodium‐ion storage. Adv. Mater. (2025). 10.1002/adma.202501779 40937944

[11] Y. He, D. Liu, J. Jiao, Y. Liu, S. He et al., Pyridinic n‐dominated hard carbon with accessible carbonyl groups enabling 98% initial coulombic efficiency for sodium‐ion batteries. Adv. Funct. Mater. **34**(39), 2403144 (2024). 10.1002/adfm.202403144

[12] J. Cui, W. Li, P. Su, X. Song, W. Ye et al., Repair surface defects on biomass derived hard carbon anodes with N‐doped soft carbon to boost performance for sodium‐ion batteries. Adv. Energy Mater. **15**(31), 2502082 (2025). 10.1002/aenm.202502082

[13] M.Q. Liu, F. Wu, Y.T. Gong, Y. Li, Y. Li et al., Interfacial-catalysis-enabled layered and inorganic-rich SEI on hard carbon anodes in ester electrolytes for sodium-ion batteries. Adv. Mater. **35**(29), 2300002 (2023). 10.1002/adma.202300002 37018163

[14] G. Huang, J. Hu, Z. Wang, F. Gao, Y. Zhou et al., Synergistic dual-engineering of hard carbons via graphene oxide nanosheets: enabling ultrahigh ice sodium-ion batteries through edge passivation and catalytic SEI design. Adv. Funct. Mater. **36**(1), e13551 (2025). 10.1002/adfm.202513551

[15] H. Li, Y. Zhou, Y. Yang, Y. Chen, Y. Zhang et al., Manipulating interphase chemistry by endogenous doping toward high-performance hard carbon anodes for sodium-ion batteries. Nano-Micro Lett. (2026). 10.1007/s40820-026-02124-9 PMC12976286 41806069

[16] C. Wu, Y. Yang, Y. Li, X. He, Y. Zhang et al., Unraveling the structure-performance relationship in hard carbon for sodium-ion battery by coupling key structural parameters. Energ. Environ. Sci. **18**(12), 6019–6031 (2025). 10.1039/d5ee00278h

[17] Y. Wan, X. Shi, Z. Zhou, W. Kuang, X. Xu et al., Ion-dipole interactions modulated anion-reinforced solvation chemistry for advanced wide-temperature sodium-ion full batteries. J. Am. Chem. Soc. **147**(33), 29939–29948 (2025). 10.1021/jacs.5c07004 40773252

[18] S.W. Zhao, F.Q. Huang, Weakly solvating few-layer-carbon interface toward high initial coulombic efficiency and cyclability hard carbon anodes. ACS Nano **18**(2), 1733–1743 (2024). 10.1021/acsnano.3c11171 38175544

[19] Y. Sun, J. Du, T. Shi, D. Zuo, J. Tian et al., Polymer-induced solid electrolyte interphase on hard carbon enabling 5C fastcharging practical sodium-ion pouch cell. Natl. Sci. Rev. **13**(4), nwag025 (2026). 10.1093/nsr/nwag025/8429798 41684699 PMC12892357

[20] G. Zhang, C. Fu, S. Gao, H. Zhao, C. Ma et al., Regulating interphase chemistry by targeted functionalization of hard carbon anode in ester-based electrolytes for high-performance sodium-ion batteries. Angew. Chem. Int. Ed. (2025). 10.1002/anie.202424028 39878445

[21] Y. Sun, D. Zuo, C. Xu, B. Peng, J.-C. Li et al., A “grafting technique” to tailor the interfacial behavior of hard carbon anode for stable sodium-ion batteries. Energ. Environ. Sci. **18**(4), 1911–1919 (2025). 10.1039/d4ee05305b

[22] P. Wang, S. Xu, S. Wang, T. Xia, J. Bai et al., Unlocking interlayer confinement enables all-slope hard carbon with ultrafast and highly reversible sodium storage. ACS Nano **19**(44), 38735–38748 (2025). 10.1021/acsnano.5c14641 41159759

[23] J. Pan, Y.H. Liu, Y.W. Sun, O. Seo, R. Kumara et al., Strategy formulation for mitigating capacity fading of Na-layered oxides. Angew. Chem. Int. Ed. **64**(22), e202503587 (2025). 10.1002/anie.202503587 40128835

[24] J. Wang, S. Li, M. Chen, C. Gao, W. Li et al., Synergistically competitive coordination for tailoring sodium cointercalation potential of graphite. Nat. Commun. **16**(1), 7628 (2025). 10.1038/s41467-025-63058-1 40817334 PMC12356933

[25] Z. Wen, R. Zhao, T. Tian, T. Zhang, X. Wang et al., Molecular stitching in polysaccharide precursor for fabricating hard carbon with ultra-high plateau capacity of sodium storage. Adv. Mater. **37**(18), 2420251 (2025). 10.1002/adma.202420251 40125840

[26] S.J. Jiang, Y.S. Xu, X.W. Sun, L. Chen, Y.N. Li et al., Lignocellulolytic bacterial engineering for tailoring the microstructure of hard carbon as a sodium-ion battery anode with fast plateau kinetics. J. Am. Chem. Soc. **147**(10), 8088–8092 (2025). 10.1021/jacs.4c15593 40021453

[27] Z. Zheng, S.J. Hu, W.J. Yin, J. Peng, R. Wang et al., CO_2_-etching creates abundant closed pores in hard carbon for high-plateau-capacity sodium storage. Adv. Energy Mater. **14**(3), 2303064 (2024). 10.1002/aenm.202303064

[28] N. LeGe, Y.H. Zhang, W.H. Lai, X.X. He, Y.X. Wang et al., Potassium escaping balances the degree of graphitization and pore channel structure in hard carbon to boost plateau sodium storage capacity. Chem. Sci. **16**(3), 1179–1188 (2024). 10.1039/d4sc04584j 39713759 PMC11660424

[29] S. Fu, G. Huang, Y. Zhou, W. Ma, Y. Chen et al., Transient NaCl templating: a scalable route to defect-rich anthracite hard carbon anodes for advanced sodium-ion batteries. Carbon **254**, 121515 (2026). 10.1016/j.carbon.2026.121515

[30] Y.L. Wang, Z.L. Yi, L.J. Xie, Y.X. Mao, W.J. Ji et al., Releasing free radicals in precursor triggers the formation of closed pores in hard carbon for sodium-ion batteries. Adv. Mater. **36**(26), 2401249 (2024). 10.1002/adma.202401249 38529803

[31] J. Zhong, S. Huang, Y. Cao, K. Pei, D. Zhang et al., Inner‐wrinkled porous carbons via soft‐hard coupling assembly strategy for ultrahigh‐capacity lithium‐ion batteries. Adv. Funct. Mater. (2025). 10.1002/adfm.202514143

[32] D. Sun, L. Zhao, P.L. Sun, K. Zhao, Y.K. Sun et al., Rationally regulating closed pore structures by pitch coating to boost sodium storage performance of hard carbon in low-voltage platforms. Adv. Funct. Mater. **34**(40), 2403642 (2024). 10.1002/adfm.202403642

[33] Q. Chen, Q. Wen, C. Li, C. Li, P. Zhao et al., High-compaction spherical carbon with tunable rich pore structures for efficient sodium storage. Adv. Mater. (2025). 10.1002/adma.202515495 41158085

[34] B. Tang, Y. Zhang, B. Ji, G. Yu, Y. Zheng et al., Ion-mediated carbon microdomain engineering boosting enhanced plateau capacity of carbon anode under high rate towards high-performance sodium dual-ion batteries. Nano-Micro Lett. **18**(1), 161 (2026). 10.1007/s40820-025-02008-4 PMC12765788 41486218

[35] Y. Wang, J. Zeng, Y. Wang, C. Zhang, Q. Wang et al., Biomass‐derived carbon with large interlayer spacing for anode of potassium ion batteries. Adv. Mater. (2024). 10.1002/adma.202410132 39428856

[36] S. You, Q. Zhang, J. Liu, Q. Deng, Z. Sun et al., Hard carbon with opened pore structure for enhanced sodium storage performance. Energy Environ. Sci. **17**(21), 8189–8197 (2024). 10.1039/d4ee02519a

[37] B. Wang, J.R. Fitzpatrick, A. Brookfield, A.J. Fielding, E. Reynolds et al., Electron paramagnetic resonance as a tool to determine the sodium charge storage mechanism of hard carbon. Nat. Commun. **15**(1), 3013 (2024). 10.1038/s41467-024-45460-3 38589362 PMC11001870

[38] J. Duan, Z. Xu, M. Li, P. Yang, H. Hu et al., Structure regulation of hard carbon with enriched semi‐closed ultramicropores for enhanced rapid sodium storage. Adv. Funct. Mater. (2025). 10.1002/adfm.202508822

[39] R. Zhou, S. Peng, Z. Wang, Y. Zhao, C. Bao et al., Dynamic sodiation‐driven pore reconstruction for superior initial‐coulombic‐efficiency and high‐rate in xylose‐based hard carbon anode. Adv. Funct. Mater. **35**(29), 2423530 (2025). 10.1002/adfm.202423530

[40] T. Zhang, T. Zhang, C. Zhao, F. Wang, L. Zhang et al., Revealing effect of aggregation structure of plant precursors on rate performance of carbon anode for sodium‐ion batteries. Adv. Funct. Mater. (2025). 10.1002/adfm.202425234

[41] S. Xu, W. Liu, S. Mao, Y. Wu, B. Yuan et al., Lotus root starch derived sustainable hard carbon for fast-charging sodium-ion batteries. Chem. Eng. J. **519**, 165014 (2025). 10.1016/j.cej.2025.165014

[42] S. Wu, J. Zhou, Q. Li, F. Wu, Natural gluten binder enabling high‐performance hard carbon anode in sodium‐ion batteries. Adv. Funct. Mater. (2025). 10.1002/adfm.202507916

[43] J. Wang, Y. Huang, W. He, Y. Li, T. Ma et al., Stepwise crosslinking-activation to create a closed pore structure of hard carbon for boosted sodium energy. J. Mater. Chem. A **13**(40), 34316–34325 (2025). 10.1039/d5ta04228c

[44] S. Li, H. Yin, Z. Xing, X. Lin, J. Liu et al., Endogenous template-directed topological engineering of lignite-derived hard carbons for kinetically accelerated sodium storage. Angew. Chem. Int. Ed. (2025). 10.1002/anie.202512936 41084268

[45] Z. Huo, H. Fang, S. Lin, J. Huang, Y. Liu et al., Atomic-scale interface engineering for robust sodium-ion battery anodes with superior stability and high energy density. Adv. Energy Mater. **15**(30), 2501288 (2025). 10.1002/aenm.202501288

[46] Z. Hou, Y. Zhao, Y. Du, F. Wu, W. He et al., Expediting sodium energy of hard carbon by cation/anion co‐interfering chemistry. Adv. Funct. Mater. (2025). 10.1002/adfm.202505468

[47] Z. Hou, W. He, F. Wu, Y. Du, F. Xu et al., Tuning π-π carbon restacking hindrance to remodify hard carbon crystallites for advanced sodium energy. Energy Storage Mater. **80**, 165014 (2025). 10.1016/j.ensm.2025.104455

[48] G. Feng, X. Yang, X. Liu, Y. Wang, Y. Xie et al., Microbially glycolysis-regulated hard carbons for sodium-ion batteries. Nano Energy **136**, 110728 (2025). 10.1016/j.nanoen.2025.110728

[49] Y. Chai, J. Guo, C. Hong, Z. Yi, W. Li et al., Reducing steric hindrance to enhance the oxidation reactivity of coal precursors for high-performance hard carbons in sodium-ion batteries. Energy Storage Mater. **83**, 104678 (2025). 10.1016/j.ensm.2025.104678

[50] J. Lin, Q. Zhou, Z. Liao, Y. Chen, Y. Liu et al., Steric hindrance engineering to modulate the closed pores formation of polymer-derived hard carbon for high-performance sodium-ion batteries. Angew. Chem. Int. Ed. **63**(39), e202409906 (2024). 10.1002/anie.202409906 38970247

[51] J.J. Liu, Y.W. You, L. Huang, Q.Z. Zheng, Z.F. Sun et al., Precisely tunable instantaneous carbon rearrangement enables low-working-potential hard carbon toward sodium-ion batteries with enhanced energy density. Adv. Mater. **36**(44), 2407369 (2024). 10.1002/adma.202407369 39221669

[52] C. Li, Q. Chen, Y. Zhang, P. Zhao, X. He et al., Molecular reconfiguration of pitch-derived hard carbon anodes with balanced thermodynamic stability and rapid sodium storage kinetics for high-performance sodium-ion batteries. Energy Storage Mater. **82**, 104580 (2025). 10.1016/j.ensm.2025.104580

[53] M. Song, Z. Hu, C. Yuan, P. Dai, T. Zhang et al., Locally curved surface with CoN_4_ sites enables hard carbon with superior sodium-ion storage performances at -40 °C. Adv. Energy Mater. **14**(23), 2304537 (2024). 10.1002/aenm.202304537

[54] H. Yu, X. Liu, Z. Zhang, Y. Wu, D. Chen et al., Engineering pseudo-graphite-dominated hard carbon as anode for sodium-ion batteries. ACS Energy Lett. **10**, 4342–4352 (2025). 10.1021/acsenergylett.5c01780

[55] S. Xu, P. Wang, B. Zhao, X. He, X. Wu, Time-sequence autogenous shrinkage engineering toward hard carbon anodes with dominant closed porosity for high-performance sodium-ion batteries. Energy Storage Mater. **88**, 105107 (2026). 10.1016/j.ensm.2026.105107

[56] H. Zhang, L. Luo, M. Guo, D. Zhang, Z. Huang et al., Biomimetic mineralization coupling with seeds-induced foaming for optimizing carbon microstructure towards ultrafast sodium ion storage. J. Power. Sources **613**, 234875 (2024). 10.1016/j.jpowsour.2024.234875

[57] B. Qiu, N. Sun, X. Li, R.A. Soomro, Y. Guan et al., Anion-anchoring and interphase manipulation for enhanced all-climate sodium storage in hard carbon anodes. Adv. Func. Mater. **35**(52), e12543 (2025). 10.1002/adfm.202512543

