# Supplementary material for: Anionic Bottom-Up Flux Orchestrated via Hard Carbon Surface Chemistry for Stable Sodium-Ion Batteries
Source: Nanomicro Lett. 2026 Sep 3;19:43. doi: 10.1007/s40820-026-02339-w (PMC13538338; doi:10.1007/s40820-026-02339-w)
Supplement: Supplementary file 1 [file 40820_2026_2339_MOESM1_ESM.docx]

Supporting Information for

**Anionic Bottom-Up Flux Orchestrated via Hard Carbon Surface Chemistry for Stable Sodium-Ion Batteries**

Peiyao Wang^1^, Shendong Xu^2,^ *, Siya Wang^1^, Xiaoyu Cui^1^, Jin Bai^1^, Yuping Sun^1^, Xuebin Zhu^1^, Bangchuan Zhao^1,^ *, Shulei Chou^3,^ *, Xingqiao Wu^3,^ *

^1^ Key Laboratory of Materials Physics, Institute of Solid State Physics, HFIPS, Chinese Academy of Sciences, Hefei 230031, P. R. China

^2^ School of Carbon Neutrality Science and Engineering, Anhui University of Science and Technology, Hefei 231131, P. R. China

^3^ Institute for Carbon Neutralization Technology, College of Chemistry and Materials Engineering, Wenzhou University, Wenzhou, Zhejiang 325035, P. R. China

* Corresponding authors. E-mail: [2024226@aust.edu.cn](mailto:2024226@aust.edu.cn) (Shendong Xu); [bczhao@issp.ac.cn](mailto:bczhao@issp.ac.cn) (Bangchuan Zhao); [chou@wzu.edu.cn](mailto:chou@wzu.edu.cn) (Shulei Chou); [xingqiaowu@wzu.edu.cn](mailto:xingqiaowu@wzu.edu.cn) (Xingqiao Wu).

**S1 Supplementary Text**

***S1.1 Materials characterization***

The morphologies of carbon samples were observed by field-emission scanning electron microscope (FE-SEM, SU 8020, HITACHI) and transmission electron microscope (TEM, Tecnai G2F20). The surface properties of samples were detected by X-ray photoelectron spectra (XPS, K-alpha, Thermo Scientific Esclab 250), and Fourier transform infrared spectroscopy (FTIR, Bruker Vertex 70). The microcrystalline structure of carbon samples was analyzed by X-ray diffraction (XRD, Philips X’pert PRO) with Cu-Kα radiation (λ=1.54 Å), Raman microscopy (Horiba HR800, Japan) with a laser wavelength of 532 nm. The specific surface areas and pore diameter distributions were exhibited by the Bruauer-Emmett-Teller (BET) measurement with N_2_ adsorption and desorption test (ASAP 2460 3.01.02) at 77 K. The graphitic nanodomain length along a-direction (*L_a_*) and the crystallinity (*L_c_*) are calculated based on Debye-Scherer equation (1) and (2), respectively.

The average *d*_(002)_ were confirmed by Bragg’s law based on the positions of the (002) peaks, as shown in the equation (3).

$L_{a}(nm)=\frac{1.84\lambda}{\beta cos\theta}$	(S1)

$L_{c}(nm)=\frac{0.89\lambda}{\beta cos\theta}$	(S2)

$d_{(002)}(nm)=\frac{\lambda}{2sin\theta}$	(S3)

Where $\lambda$ is wavelength of X-ray beam, 2*θ* is diffraction angle, *β* refers to full width at half maximum (FWHM) value of different peaks.

Time-of flight secondary ion mass spectrometry was performed with a TOF-SIMS (ION-TOF, TOF.SIMS5). The atomic force microscopy (AFM) system (Bruker Dimension Icon) was employed to collect the topography and modulus images of the post-cycled electrodes.

***S1.2 Correlated Calculations***

The density functional theory (DFT) calculations were carried out using Gaussian 16W. All structures were calculated using the B3LYP functional in combination with the 6-311G(d,p) basis set,[S1] and DFT-D3 correction was employed in the calculations to ensure the authenticity of weak interactions.[S2, S3] The differential charges and frontier orbitals were analyzed using the Multiwfn software (Multiwfn_3.8_dev_bin_Win64), and the frontier orbitals were visualized using VMD (Version 1.9.3).

The differential charges were visualized using the VESTA software. The molecular dynamics (MD) simulations of hard carbon-electrolyte interface were conducted using the GROMACS simulation package (version 2024) with the GAFF force field.[S4, S5] Three types of amorphous hard carbon models with different surface functional groups (oxygen-containing, nitrogen-containing, and oxygen-nitrogen containing groups) were constructed in-house. The hard carbon structures were kept rigid during the simulations with a density of approximately 0.5 g cm^-3^. The electrolyte solution consisting of 1 M NaPF_6_ in diglyme was randomly filled into the void space of the hard carbon matrix.

All force field parameters were obtained from the GAFF force field.[S6] The RESP atomic charges for all species were calculated using the Multiwfn program based on density functional theory (DFT) calculations at the B3LYP/6-31G(d) level using Gaussian 16 software.[S1] (Noted: The calculated adsorption energies are obtained for gas-phase clusters without explicit solvent. While solvation effects may reduce the absolute values under operating conditions, the relative ordering and functional differentiation among different sites remain robust and provide meaningful mechanistic insight.) The simulation box was constructed as a cubic box with dimensions of approximately 5.5 nm, with periodic boundary conditions applied in all three directions. All simulations used an MD timestep of 1 fs. The Particle Mesh Ewald (PME) algorithm was employed to calculate the long-range electrostatic interactions with a cut-off of 1.6 nm for short-range interactions, while the cut-off for van der Waals interactions was also set to 1.6 nm. The temperature was controlled using the V-rescale thermostat.[S7]

The systems were first energy-minimized using the steepest descent algorithm until convergence was achieved. Subsequently, the systems were equilibrated in the canonical (NVT) ensemble at 298.15 K for 10 ns to ensure proper relaxation of the electrolyte within the hard carbon matrix. Finally, production runs in the NVT ensemble at 298.15 K for 40 ns were performed for data collection and analysis.[S8, S9]

***S1.3 Detailed construction of the amorphous hard carbon models***

The O-HC, N-HC, and N/O-HC amorphous hard carbon models were constructed using Materials Studio, where a graphitized carbon backbone consisting predominantly of aromatic carbon atoms was first established, and heteroatoms were subsequently introduced to generate the three distinct model types. Oxygen-containing functional groups, including hydroxyl and carbonyl/epoxy groups, were grafted onto edge and defect sites of the carbon framework following the functionalization conventions of oxidized graphene, while nitrogen-containing groups such as pyridinic‑N, pyrrolic‑N, and graphitic‑N were incorporated in accordance with the bonding configurations identified from experimental XPS characterization. After the initial atomic structures were assembled, each single‑molecule model was subjected to geometry optimization at the B3LYP/6‑31G* level using the Gaussian software package to yield a physically reasonable starting configuration. Force field parameters and RESP partial charges were subsequently assigned to all atoms using the Sobtop program based on the GAFF force field, generating the topology files required for GROMACS‑based molecular dynamics simulations. Thirty such molecules were placed in a cubic periodic simulation box with edge lengths of 55.219 × 55.219 × 55.219 Å^3^, giving a total of 6030 hard carbon atoms in the supercell and an estimated model density of approximately 0.50 g cm^-3^. All functional groups are distributed randomly across edge and defect sites of the carbon skeleton to reflect the disordered nature of real amorphous hard carbon materials. For the interfacial simulations with the electrolyte, 30 hard carbon molecules were packed together with 210 diglyme solvent molecules, 30 sodium cations, and 30 hexafluorophosphate anions into an orthorhombic periodic box of dimensions 48.89 × 49.00 × 58.37 Å using the PACKMOL software, producing a full simulation system of approximately 11,676 atoms.

***S1.4 Stabilization protocol for the models***

The stable equilibrium configuration was obtained through a three‑stage protocol. The system was first subjected to energy minimization using the steepest descent algorithm for up to 5000 steps with a convergence threshold of 500 kJ mol^-1^ nm^-1^ and a step size of 0.001, during which all hard carbon framework atoms were frozen in all three spatial dimensions to eliminate unphysical atomic overlaps in the initial configuration while allowing the electrolyte molecules to relax freely. The system was then equilibrated under the NVT ensemble at 298.15 K for 10 ns using a time step of 1 fs, with temperature controlled by a V‑rescale thermostat with a coupling time constant of 0.1 ps, and with the hard carbon atoms remaining frozen throughout. Long‑range electrostatic interactions were handled by the particle mesh Ewald method with a real‑space cutoff of 1.3 nm, van der Waals interactions were treated with a cutoff scheme at 1.3 nm, and long‑range dispersion corrections were applied to both energy and pressure. After equilibration, a 20 ns production run was performed under identical NVT conditions, with trajectories saved every 2 ps for subsequent analysis of radial distribution functions, ionic diffusion coefficients, and interfacial adsorption structures. This sequential procedure of energy minimization, NVT equilibration, and production dynamics ensured that a fully relaxed and thermodynamically stable interfacial configuration was obtained before any structural or dynamic properties were extracted.

**S2 Supporting Figures**

**Fig. S1** (a) Snapshots of the structure of electrolyte. (b, c) Solvation structure of Na+ with PF_6_^-^ and G2 molecule calculated from MD simulations.

**Fig. S2** Charge distribution image of oxygen-containing functional groups adsorbing G2 molecular and NaPF_6_.

**Fig. S3** Charge distribution image of nitrogen-containing functional groups adsorbing G2 molecular and NaPF_6_

**Fig. S4** The G2 and PF_6_^-^ adsorption energy of pyrrolic-N/C=O and pyridinic-N/C=O.

**Fig. S5** LUMO energy level of different functional types with electrolyte.

**Fig. S6** HOMO/LUMO energy of NaPF_6_ and G2 molecular.

**Fig. S7** HOMO energy level of different functional types with electrolyte.

**Fig. S8** The Na^+^ adsorption energy of different groups.

**Fig. S9** Na^+^ adsorption energy of pyrrolic-N/C=O and pyridinic-N/C=O.

**Fig. S10** (a) FTIR curves of pre-PS and PS. (b) TG curves of PS.

**Fig. S11** TEM images of P-HC and R-HC.

**Fig. S12** (a, d) HRTEM of R-HC and P-HC. (b, e) Graph of graphite-like interlayer distance measurement data based on R-HC and P-HC. (c, f) Schematic of Na^+^ diffusion path within R-HC and P-HC.

**Fig. S13** Comparison of *d_(002)_*, *L_a_*, and *L_c_*.

**Fig. S14** XPS spectra of R-HC and P-HC.

**Fig. S15** High-resolution (a) C 1s spectra, (b) O 1s spectra, (c) N 1s pattern of samples, and (d-f) corresponding contents of different C, O, and N species in R-HC and P-HC.

**Fig. S16** FTIR patterns.

**Fig. S17** Pore size distributions of R-HC and P-HC tested by N_2_ adsorption-desorption.

**Fig. S18** Small-angle X-ray scattering patterns of R-HC and P-HC.

**Fig. S19** Comparison of the plateau and sloping capacity of electrodes based on the 2nd discharge/charge capacity.

**Fig. 20** (a-c) Three parallel electrodes of R-HC tested under the same test conditions (0.05 A g^-1^)

**Fig. S21** Initial three CV plots of (a) R-HC and (b) P-HC at scan rates of 0.1 mV s^-1^.

**Fig. S22** GCD plots of (a) R-HC and (b) P-HC at different current density.

**Fig. S23** EIS plots of R-HC and P-HC (a) before and (b) after 100 cycles.

**Fig. S24** GITT measurements performed at a pulse current of 0.05 A g^-1^.

**Fig. S25** (a, c) Multi-rate scan CV curves of R-HC and P-HC, (b, d) linear relationship of log (*i*) versus log (*v*) of peaks at 0.01 V and 0.1 V for R-HC and P-HC.

**Fig. S26** Selective EIS plots of (a) P-HC, and (b) R-HC at different charge state.

**Fig. S27** DRT plots of P-HC during first cycle.

**Fig. S28** Ex-situ XRD of R-HC during first cycle.

**Fig.S29** Digital photo of R-HC electrodes at different sodiation state soaked in ethanol solution with 1% phenolphthalein.

**Fig. S30** Theoretical calculations of PF_6_^-^ decomposition energy barriers in (a) O-HC, (b) N-HC, (c) N/O-HC models, and (d) corresponding decomposition energy.

**Fig. S31** Ex-situ XPS of (a) R-HC, and (b) P-HC electrodes discharged at 0.6, 0.3 and 0.01 V.

**Fig. S32** The proportion of SEI components calculated from the (a) C 1s, (b) O 1s, and (c) F 1s spectra.

**Fig. S33** Depth-profiling XPS spectra of O 1s spectra of (a) P-HC, and (b) R-HC.

**Fig. S34** 3D reconstruction models illustrating the distribution of PF_6_^-^, NaF^-^, C_2_H_3_O^-^ obtained from TOF-SIMS of the SEI layer formed on P-HC and R-HC.

**Fig. S35** TOF-SIMS depth profiles of (a) NaF_2_^-^, (b) NaO^-^, (c) CF^-^, (d) PF_6_^-^, (e) NaF^-^, (f) C_2_H_3_O^-^.

**Fig. S36** Surface species (PF_6_^-^, NaF^-^, C_2_H_3_O^-^) distributions of P-HC and R-HC.

**Fig. S37** SEM images of (a) R-HC and (b) P-HC after 500 cycles.

**Fig. S38** HRTEM images of (a) R-HC and (b) P-HC electrodes after 500 cycles.

**Fig. S39** The initial three cycles of NFPP.

**Table S1** The ratios of different solvation structures in the O-HC

| Solvation structures | Quantity | Ratios (%) |
| --- | --- | --- |
| G2(1)HC_s_(1)Na(1) | 330 | 10.89 |
| G2(1)Na(1)PF_6_(1) | 294 | 9.71 |
| G2(2)HC_s_(1)Na(1) | 289 | 9.54 |
| G2(3)Na(1)PF_6_(1) | 288 | 9.51 |
| G2(3)HC_s_(1)Na(1) | 272 | 8.98 |
| G2(1)HC_s_(1)Na(1)PF_6_(1) | 249 | 8.22 |
| G2(2)Na(1)PF_6_(1) | 226 | 7.46 |
| G2(1)Na(1)PF_6_(2) | 136 | 4.49 |
| G2(1)Na(1) | 114 | 3.76 |
| G2(1)HC_s_(2)Na(1) | 112 | 3.7 |
| Na(1)PF_6_(2) | 104 | 3.43 |
| HC_s_(1)Na(1)PF_6_(2) | 101 | 3.33 |
| HC_s_(1)Na(1)PF_6_(1) | 101 | 3.33 |
| Na(1)PF_6_(1) | 101 | 3.33 |
| G2(2)HC_s_(2)Na(1) | 90 | 2.97 |
| HC_s_(2)Na(1) | 87 | 2.87 |
| G2(1)HC_s_(1)Na(1)PF_6_(2) | 62 | 2.05 |
| G2(2)HC_s_(1)Na(1)PF_6_(1) | 53 | 1.75 |
| HC_s_(1)Na(1) | 15 | 0.5 |
| G2(2)Na(1) | 4 | 0.13 |
| G2(2)Na(1)PF_6_(2) | 1 | 0.03 |
| total | 3029 | 100 |

HCs: represent the all structures of the HC surface involved in the solvation process.

**Table S2** The ratios of different solvation structures in the N-HC

| Solvation structures | Quantity | Ratios (%) |
| --- | --- | --- |
| G2(1)Na(1)PF_6_(1) | 636 | 21.03 |
| Na(1)PF_6_(1) | 420 | 13.89 |
| G2(2)Na(1)PF_6_(1) | 398 | 13.16 |
| G2(3)Na(1)PF_6_(1) | 363 | 12 |
| G2(1)HC_s_(1)Na(1) | 248 | 8.2 |
| G2(1)HC_s_(1)Na(1)PF_6_(1) | 162 | 5.36 |
| G2(1)Na(1) | 135 | 4.46 |
| Na(1) | 120 | 3.97 |
| G2(2)Na(1) | 120 | 3.97 |
| HC_s_(1)Na(1) | 102 | 3.37 |
| Na(1)PF_6_(2) | 101 | 3.34 |
| HC_s_(1)Na(1)PF_6_(1) | 100 | 3.31 |
| G2(3)Na(1) | 80 | 2.65 |
| G2(2)HC_s_(1)Na(1)PF_6_(1) | 39 | 1.29 |
| Total | 3024 | 100 |

**Table S3** The ratios of different solvation structures in the N/O-HC

| Solvation structures | Quantity | Ratios (%) |
| --- | --- | --- |
| G2(2)Na(1)PF_6_(1) | 389 | 12.85 |
| G2(2)HCs(1)Na(1) | 353 | 11.66 |
| G2(1)Na(1)PF_6_(1) | 350 | 11.56 |
| G2(1)Na(1)PF_6_(2) | 349 | 11.53 |
| G2(3)HC_s_(1)Na(1) | 309 | 10.2 |
| G2(1)Na(1) | 209 | 6.9 |
| G2(1)HC_s_(1)Na(1) | 175 | 5.78 |
| G2(1)HC_s_(1)Na(1)PF_6_(1) | 170 | 5.61 |
| Na(1)PF_6_(2) | 156 | 5.15 |
| Na(1) | 110 | 3.63 |
| Na(1)PF_6_(1) | 102 | 3.37 |
| G2(3)Na(1) | 95 | 3.14 |
| G2(2)HCs(2)Na(1) | 93 | 3.07 |
| G2(3)Na(1)PF_6_(1) | 68 | 2.25 |
| G2(2)Na(1) | 59 | 1.95 |
| HC_s_(1)Na(1)PF_6_(1) | 32 | 1.06 |
| G2(1)HC_s_(2)Na(1) | 8 | 0.26 |
| HC_s_(1)Na(1) | 1 | 0.03 |
| Total | 3028 | 100 |

**Note to Table S1-S3:** To gain deeper statistical insight into this interfacial reorganization, the population distribution of solvation structures was analyzed (Table S1-S3). The N-O co-functionalized surface exhibits a distinct solvation structure, which containing two PF_6_^-^ anions (e.g., G2(1)Na(1)PF_6_(2)) are significantly enriched, with a population of 11.53% compared to only 3.34% and 4.49% on the O-rich and N-rich surfaces, respectively, indicating a localized anion-rich environment at the interface. Besides, solvation structures involving direct contact with the surface functional groups (HC_s_) become predominant, with key configurations like G2(2)HC_s_(1)Na(1) reaching 11.66%. These structural changes collectively demonstrate a substantial redistribution of interfacial species, where PF_6_^-^ anions become more prevalent in the Na^+^ solvation shell while nitrogen sites become less accessible to Na^+^.[S10] This redistribution signifies a fundamental functional shift of nitrogen sites from primarily coordinating cations to engaging with anions, creating an ideal precursor environment for subsequent anion-preferential adsorption and decomposition.[S11] Collectively, these simulation results provide a molecular-scale portrait of the optimized bottom-up anionic transfer dynamics, wherein anions are selectively recruited from the electrolyte bulk and enriched at the inner Helmholtz plane, establishing the necessary concentration gradient for directed interfacial reactions.

**Table S4** A comparative analysis of the hard carbon’s electrochemical performance among reported studies (All the applied electrolyte in these works was 1 M NaPF_6_ in diglyme).

| Anode | Current density (A g^-1^) | Specific capacity (mAh g^-1^) | Ref. |
| --- | --- | --- | --- |
| CoHC | 5 | 176.3 | [S12] |
| HC-PK-6 | 3.2 | 70 | [S13] |
| S1400 | 1 | 292.3 | [S14] |
| HC-GT | 1 | 226.2 | [S15] |
| HC-P | 2 | 106.3 | [S16] |
| FHC | 2 | 215 | [S17] |
| HCL-Na | 5 | 125 | [S18] |
| BLBWC | 1 | 272 | [S19] |
| Mn-PHCS | 5 | 166 | [S20] |
| ca-HC | 2 | 221.6 | [S21] |
| HC-CA | 1 | 152.7 | [S22] |
| YHCs-G | 6 | 134.7 | [S23] |
| ISO-PC | 1 | 90.2 | [S24] |
| R-HC | 1/2/5/10 | 265.2/235.1/188.5/162.1 | This work |

**Supplementary References**

[S1] M. P. Andersson, P. Uvdal, New scale factors for harmonic vibrational frequencies using the b3lyp density functional method with the triple- basis set 6-311+g(d,p). J. Phys. Chem. A **109**(2937-2941 (2005). https://doi.org/10.1021/jp045733a

[S2] T. D. Kühne, M. Iannuzzi, M. Del Ben, V. V. Rybkin, P. Seewald et al., Cp2k: An electronic structure and molecular dynamics software package - quickstep: Efficient and accurate electronic structure calculations. J. Chem. Phys. **152**(19), 194103 (2020). <https://doi.org/10.1063/5.0007045>

[S3] J. VandeVondele, M. Krack, F. Mohamed, M. Parrinello, T. Chassaing et al., Quickstep: Fast and accurate density functional calculations using a mixed gaussian and plane waves approach. Computer Phys. Commun. **167**(2), 103-128 (2005). <https://doi.org/10.1016/j.cpc.2004.12.014>

[S4] T. W. Surta, E. Koh, Z. F. Li, D. B. Fast, X. L. Ji et al., Combining experimental and theoretical techniques to gain an atomic level understanding of the defect binding mechanism in hard carbon anodes for sodium ion batteries. Adv. Energy Mater. **12**(25), 2200647 (2022). <https://doi.org/10.1002/aenm.202200647>

[S5] H. J. C. Berendsen, D. Vanderspoel, R. Vandrunen, Gromacs - a message-passing parallel molecular-dynamics implementation. Computer Phys. Commun. **91**(1-3), 43-56 (1995). <https://doi.org/10.1016/0010-4655(95)00042-e>

[S6] Y. Duan, C. Wu, S. Chowdhury, M. C. Lee, G. M. Xiong et al., A point-charge force field for molecular mechanics simulations of proteins based on condensed-phase quantum mechanical calculations. J. Computational Chem. **24**(16), 1999-2012 (2003). <https://doi.org/10.1002/jcc.10349>

[S7] M. F. Crowley, T. A. Darden, T. E. Cheatham, D. W. Deerfield, Adventures in improving the scaling and accuracy of a parallel molecular dynamics program. J. Supercomputing **11**(3), 255-278 (1997). <https://doi.org/10.1023/a:1007907925007>

[S8] W. Li, Y. Duan, S. Ge, W. Wu, K. Song et al., Locking-chain electrolyte additive enabling moisture-tolerant electrolytes for sodium-ion batteries. Nat. Commun. **16**(1), 6405 (2025). <https://doi.org/10.1038/s41467-025-61603-6>

[S9] W. D. Cornell, P. Cieplak, C. I. Bayly, I. R. Gould, K. M. Merz et al., A second generation force field for the simulation of proteins, nucleic acids, and organic molecules. J. Am. Chem. Soc. **118**(9), 2309-2309 (1996). <https://doi.org/10.1021/ja955032e>

[S10] B. Song, Z. Luo, X. Liu, Y. Peng, J. Wang et al., Bridging donor ligands enable an ultrastable graphite anode for sodium-ion batteries. Angew. Chem. Int. Ed. e202503027 (2025). <https://doi.org/10.1002/anie.202503027>

[S11] Y. Yang, Y. Yang, Y. Yang, Y. Yao, H. Yang et al., Advanced electrolyte engineering for low-temperature sodium-ion batteries. Adv. Mater. e13868 (2025). <https://doi.org/10.1002/adma.202513868>

[S12] R. Zhou, S. Peng, Z. Wang, Y. Zhao, C. Bao et al., Dynamic sodiation‐driven pore reconstruction for superior initial‐coulombic‐efficiency and high‐rate in xylose‐based hard carbon anode. Adv. Funct. Mater. **35**(29), 2423530 (2025). <https://doi.org/10.1002/adfm.202423530>

[S13] T. Zhang, T. Zhang, C. Zhao, F. Wang, L. Zhang et al., Revealing effect of aggregation structure of plant precursors on rate performance of carbon anode for sodium‐ion batteries. Adv. Funct. Mater. 2425234 (2025). <https://doi.org/10.1002/adfm.202425234>

[S14] S. Xu, W. Liu, S. Mao, Y. Wu, B. Yuan et al., Lotus root starch derived sustainable hard carbon for fast-charging sodium-ion batteries. Chem. Eng. J. **519**(165014 (2025). <https://doi.org/10.1016/j.cej.2025.165014>

[S15] S. Wu, J. Zhou, Q. Li, F. Wu, Natural gluten binder enabling high‐performance hard carbon anode in sodium‐ion batteries. Adv. Funct. Mater. 2507916 (2025). <https://doi.org/10.1002/adfm.202507916>

[S16] J. Wang, Y. Huang, W. He, Y. Li, T. Ma et al., Stepwise crosslinking–activation to create a closed pore structure of hard carbon for boosted sodium energy. J. Mater. Chem. A **13**(40), 34316-34325 (2025). <https://doi.org/10.1039/d5ta04228c>

[S17] Y. Sun, D. Zuo, C. Xu, B. Peng, J.-C. Li et al., A "grafting technique" to tailor the interfacial behavior of hard carbon anode for stable sodium-ion batteries. Energ. Environ. Sci. **18**(4), 1911-1919 (2025). <https://doi.org/10.1039/d4ee05305b>

[S18] S. Li, H. Yin, Z. Xing, X. Lin, J. Liu et al., Endogenous template-directed topological engineering of lignite-derived hard carbons for kinetically accelerated sodium storage. Angew. Chem. Int. Ed. e202512936 (2025). <https://doi.org/10.1002/anie.202512936>

[S19] S. J. Jiang, Y. S. Xu, X. W. Sun, L. Chen, Y. N. Li et al., Lignocellulolytic bacterial engineering for tailoring the microstructure of hard carbon as a sodium-ion battery anode with fast plateau kinetics. J. Am. Chem. Soc. **147**(10), 8088-8092 (2025). <https://doi.org/10.1021/jacs.4c15593>

[S20] G. Huang, J. Hu, Z. Wang, F. Gao, Y. Zhou et al., Synergistic dual‐engineering of hard carbons via graphene oxide nanosheets: Enabling ultrahigh ice sodium-ion batteries through edge passivation and catalytic sei design. Adv. Funct. Mater. **36**(1), e13551 (2025). <https://doi.org/10.1002/adfm.202513551>

[21] Z. Hou, Y. Zhao, Y. Du, F. Wu, W. He et al., Expediting sodium energy of hard carbon by cation/anion co‐interfering chemistry. Adv. Funct. Mater. 2505468 (2025). <https://doi.org/10.1002/adfm.202505468>

[S22] Z. Hou, W. He, F. Wu, Y. Du, F. Xu et al., Tuning π-π carbon restacking hindrance to remodify hard carbon crystallites for advanced sodium energy. Energy Storage Mater. **80**, 165014 (2025). <https://doi.org/10.1016/j.ensm.2025.104455>

[S23] G. Feng, X. Yang, X. Liu, Y. Wang, Y. Xie et al., Microbially glycolysis-regulated hard carbons for sodium-ion batteries. Nano Energy **136**(110728 (2025). <https://doi.org/10.1016/j.nanoen.2025.110728>

[S24] Y. Chai, J. Guo, C. Hong, Z. Yi, W. Li et al., Reducing steric hindrance to enhance the oxidation reactivity of coal precursors for high-performance hard carbons in sodium-ion batteries. Energy Storage Mater. **83**, 104678 (2025). <https://doi.org/10.1016/j.ensm.2025.104678>
